# Temperature‐mediated acquisition of rare heterologous symbionts promotes survival of coral larvae under ocean warming

**DOI:** 10.1111/gcb.16057

**Published:** 2022-01-05

**Authors:** Shayle B. Matsuda, Leela J. Chakravarti, Ross Cunning, Ariana S. Huffmyer, Craig E. Nelson, Ruth D. Gates, Madeleine J. H. van Oppen

**Affiliations:** ^1^ Hawai‘i Institute of Marine Biology University of Hawai‘i at Mānoa Kāne‘ohe Hawai‘i USA; ^2^ Australian Institute of Marine Science Townsville Queensland Australia; ^3^ Daniel P. Haerther Center for Conservation and Research John G. Shedd Aquarium Chicago Illinois USA; ^4^ Department of Biological Sciences University of Rhode Island Kingston Rhode Island USA; ^5^ Daniel K. Inouye Center for Microbial Oceanography: Research and Education Department of Oceanography and Sea Grant College Program University of Hawai‘i at Mānoa Honolulu Hawai‘i USA; ^6^ School of BioSciences The University of Melbourne Parkville Victoria Australia

**Keywords:** climate change, coral, digital droplet PCR (ddPCR), larvae, Symbiodiniaceae, symbiont choice experiments, symbiosis, thermal stress

## Abstract

Reef‐building corals form nutritional symbioses with endosymbiotic dinoflagellates (Symbiodiniaceae), a relationship that facilitates the ecological success of coral reefs. These symbionts are mostly acquired anew each generation from the environment during early life stages (“horizontal transmission”). Symbiodiniaceae species exhibit trait variation that directly impacts the health and performance of the coral host under ocean warming. Here, we test the capacity for larvae of a horizontally transmitting coral, *Acropora tenuis*, to establish symbioses with Symbiodiniaceae species in four genera that have varying thermal thresholds (the common symbiont genera, *Cladocopium* and *Durusdinium*, and the less common *Fugacium* and *Gerakladium*). Over a 2‐week period in January 2018, a series of both no‐choice and four‐way choice experiments were conducted at three temperatures (27, 30, and 31°C). Symbiont acquisition success and cell proliferation were measured in individual larvae. Larvae successfully acquired and maintained symbionts of all four genera in no‐choice experiments, and >80% of larvae were infected with at least three genera when offered a four‐way choice. Unexpectedly, *Gerakladium* symbionts increased in dominance over time, and at high temperatures outcompeted *Durusdinium*, which is regarded as thermally tolerant. Although *Fugacium* displayed the highest thermal tolerance in culture and reached similar cell densities to the other three symbionts at 31°C, it remained a background symbiont in choice experiments, suggesting host preference for other symbiont species. Larval survivorship at 1 week was highest in larvae associated with *Gerakladium* and *Fugacium* symbionts at 27 and 30°C, however at 31°C, mortality was similar for all treatments. We hypothesize that symbionts that are currently rare in corals (e.g., *Gerakladium*) may become more common and widespread in early life stages under climate warming. Uptake of such symbionts may function as a survival strategy in the wild, and has implications for reef restoration practices that use sexually produced coral stock.

## Graphical abstract

Acquiring algal symbionts is critical for the survival of coral during early life stages. *Acropora tenuis* larvae successfully acquired rare thermally tolerant algal symbionts, and symbiont identity influenced survival under elevated temperatures. At elevated temperatures, rare heterologous *Fugacium kawagutii* in the no‐choice treatment had the highest survivorship. In the four‐way choice treatment (symbiont competition), *Gerakladium* sp. was the most prevalent and dominant, exhibiting the highest relative abundances of homologous or heterologous symbionts.

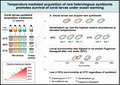

## 1 INTRODUCTION

Coral bleaching events are increasing in severity, duration, and frequency globally with recovery windows shortening between heating events; for example, Australia's Great Barrier Reef in 2016, 2017, and 2020 (Hughes et al., 2019; Thiault et al., 2020), Kāneʻohe Bay, O‘ahu, Hawai‘i in 2014, 2015, and 2019 (Matsuda et al., 2020; Ritson‐Williams & Gates, 2020), Mo'orea, French Polynesia in 2002 (Penin et al., 2007) and 2019 (Burgess et al., 2021), and the Florida Keys in 2014 and 2015 (Fisch et al., 2019). In order to replenish reefs following mortality events, the recovery and persistence of reefs depend on reproduction, recruitment, and survival of new coral offspring (Doropoulos et al., 2015). However, bleaching compromises the reproductive capacity of surviving corals (Baird & Marshall, 2002; Howells et al., 2016; Johnston et al., 2020; Ward et al., 2002), making the survival of available offspring even more critical for reef persistence (Hughes et al., 2019; Ward et al., 2002). Growth and survival of early coral life stages through the recruitment bottleneck is contingent upon the successful establishment of symbiosis with algal endosymbionts (family: Symbiodiniaceae) that provide coral nutrition through photosynthetically fixed carbon (Aranda et al., 2016; Harii et al., 2010). However, the influence of thermal stress on the timing and success of establishing symbiosis and subsequent proliferation of the symbiont population within the host is not well understood.

Corals acquire their algal symbionts through three strategies: vertical transmission: directly from the parent colony; horizontal transmission: from the surrounding environment (Cumbo et al., 2013); or mixed‐mode transmission: a combination of the two (Quigley et al., 2018). Horizontal transmission is the most widespread mode of acquisition for reef‐building corals (Baird et al., 2009; Fadlallah, 1983), and symbioses are established either in larval or post‐settlement stages (i.e., recruits). Coral larvae primarily utilize endogenous energy reserves (i.e., lipids) during development, however, symbionts may provide an additional source of nutrition (Harii et al., 2007). Associating with algal symbionts at the larval stage can have fitness consequences, such as a higher settlement rate in *Lobactis scutaria* (Schwarz et al., 1999) and greater survival in *Acropora yongei* (Suzuki et al., 2013). However, these effects are variable and species specific—for example, establishment of symbiosis had no effect on settlement in *Platygyra acuta* (Ng et al., 2019), and even reduced survival in *Orbicella faveolata* larvae (Hartmann et al., 2018).

Of the currently described genera of Symbiodiniaceae (LaJeunesse et al., 2018, 2021; Nitschke et al., 2020; Pochon & LaJeunesse, 2021), *Symbiodinium*, *Breviolum*, *Cladocopium,* and *Durusdinium* are most commonly associated with reef‐building corals (Baker, 2003). Although most corals associate with one or sometimes two dominant symbionts, additional species, in the same or different genera, may be present at background levels (<5%–10%). As molecular techniques have achieved higher resolution, symbionts from less common genera have recently been identified in low proportions as subdominant or background coral symbionts in some species (Boulotte et al., 2016; Cunning et al., 2015; Qin et al., 2019; Stat et al., 2013). For example, *Gerakladium* has been found with *Stylophora pistillata* (van Oppen et al., 2005), *Merulina ampliata* (G3: 2.3% relative abundance), *Platygyra daedalea* (G3: 3.3% relative abundance), *Porites lutea* (G3: 1.9% relative abundance), and *Hydnophora exesa* (Qin et al., 2019). *Fugacium* is the dominant symbiont in only a single hermatypic coral, *Alveopora japonica* (F‐Ajap) (De Palmas et al., 2015), and has also been found in background symbioses, for example, with *P. lutea* (<0.1% relative abundance; Qin et al., 2019) (Teschima et al., 2019). However, the influence of background symbionts on holobiont performance is still largely unknown.

Symbiodiniaceae species display differential functional performance and nutritional characteristics, and therefore it is critically important to understand how symbiosis dynamics impact survival during coral early life history. Symbiodiniaceae provide photosynthetic products to the host (Muscatine & Cernichiari, 1969), but the quality and quantity of translocated photosynthates varies among symbiont species, which can have important implications for performance (Cantin et al., 2009; Stat et al., 2008). Coral flexibility in algal symbiont association is a potentially beneficial trait, allowing the host to respond to the current environmental conditions by selecting the most appropriate symbiont(s); however, there may also be negative impacts to the host. Association with *Durusdinium* could be an adaptive response to warming oceans because *Durusdinium* species often confer greater thermal tolerance to their hosts than *Cladocopium* (Berkelmans & van Oppen, 2006; Glynn et al., 2001; Manzello et al., 2019; Rowan, 2004; Silverstein et al., 2015; however, see Abrego et al., 2008). For example, *Montipora capitata* associates with both *Cladocopium* and *Durusdinium* symbionts (Stat et al., 2011) and exhibits physiological trade‐offs between these partners (Wall et al., 2020). *Durusdinium* confers higher coral thermal tolerance on its host, but it is also known to provide less nutrition (carbon translocation) to the host than *Cladocopium* (Cantin et al., 2009), which may affect host performance during ambient temperature conditions (Jones & Berkelmans, 2010, 2011; Wall et al., 2020).

There is evidence of symbiotic flexibility in larval and recruit stages, where early life stages have been found to associate with more or different types of symbionts than adults (Abrego et al., 2009b; Gómez‐Cabrera et al., 2008; Little et al., 2004). For example, *Acropora tenuis* larvae and juveniles can be dominated by *Durusdinium*, *Cladocopium,* or a mixture, despite most adult colonies being dominated by *Cladocopium* (Little et al., 2004; Yamashita et al., 2021). Recently, low levels of *Fugacium* sp. were found in *A. tenuis* juveniles, which has not yet been observed in adults (Quigley et al., 2016). After symbionts are incorporated into the host's gastrodermal cells (i.e., “infection”), there is a “winnowing” period during which the symbiont community stabilizes and reflects adult symbiotic associations (Gómez‐Cabrera et al., 2008; Rodriguez‐Lanetty et al., 2006). Early life history symbiosis flexibility therefore provides a window of opportunity for symbiotic flexibility to provide survival advantages. It is critical to understand how symbiotic flexibility in early life stages is affected by, and mediates response to, climate change driven ocean warming, and whether rare symbionts may present benefits and/or trait trade‐offs.

In laboratory settings, inoculation experiments have evaluated the capacity for different symbionts to associate with and proliferate within coral larvae (Bay et al., 2011; Cumbo et al., 2013; Schwarz et al., 1999). The majority of these studies were no‐choice experiments where corals were exposed to a single symbiont species (Rodriguez‐Lanetty et al., 2006; Schwarz et al., 1999; Weis et al., 2001; Yakovleva et al., 2009) and demonstrated that symbionts found naturally in symbiosis with adult corals (i.e., homologous) have greater infectivity and proliferation than those that are not naturally dominant (i.e., heterologous). Multi‐genus infections can provide further insights on symbiont infection under competition and have been studied in other cnidarian‐Symbiodiniaceae models, including sea anemones (Gabay et al., 2019; Herrera et al., 2020) and an octocoral (McIlroy et al., 2019); however, no studies to date have examined infection dynamics of more than two Symbiodiniaceae genera in scleractinian corals.

Association with rare symbionts with enhanced thermal thresholds could be a product of host adaptive responses, competition among symbionts, or early life history acquisition of symbionts that are maintained at low abundances. The use of next generation sequencing has resulted in an increase in our capacity to identify rare background symbionts in corals (Silverstein et al., 2012), and has provided evidence of symbiont assemblages changing (i.e., “switching” or “shuffling”) in response to heat stress (Boulotte et al., 2016; Cunning et al., 2018; Rouzé et al., 2017; but see Stat et al., 2009). Although rare, there is evidence that coral‐Symbiodiniaceae partnerships change when a new symbiont becomes available. *Durusdinium trenchii* was recently introduced into the Caribbean and there has been an increase in dominance of this symbiont species over the past few decades in many coral species in the Caribbean (e.g., species here; Pettay et al., 2015). *Durusdinium trenchii* confers increased bleaching resistance to the host over conspecifics associated with historic partners (Manzello et al., 2019; Rowan et al., 1997). Coral–algal associations in adult corals also vary across environmental gradients, including marginal and extreme environments. For example, *Goniastrea aspera*, which associates with *Cladocopium* in “optimal” sites, forms symbiosis with *Durusdinium* in mangroves, characterized by high temperature and light conditions as well as high turbidity (Hennige et al., 2010). These differences in symbiont assemblages by environment may also impact fitness and performance under future climate change related stressors. Given variation in symbiotic relationships across environmental conditions, it is critical to evaluate how the environment drives symbiosis formation in early life stages.

Here, we investigated symbiont infection dynamics in four‐way choice and no‐choice experiments and tracked implications for coral larval survival under thermal stress. We hypothesized that larvae exposed to higher temperatures would (1) associate with a greater proportion of thermally tolerant *Durusdinium* than thermally sensitive *Cladocopium*, and (2) exhibit reduced algal cell proliferation over time accompanied by higher larval mortality. We exposed larvae of *A. tenuis*, a broadcast spawner and horizontal transmitter, to four species of Symbiodiniaceae in no‐choice and four‐way choice experiments over a 14 day period at 27, 30, and 31°C. *Acropora tenuis* is a common reef‐building coral on the Great Barrier Reef, whose adult colonies mostly associate with Symbiodiniaceae in the genus *Cladocopium* (van Oppen et al., 2001). Juveniles can associate with *Symbiodinium*, *Cladocopium* (C1 predominantly, and other C‐types), and/or *Durusdinium* (D, D1, D1a, and other D‐types) (Little et al., 2004; Quigley et al., 2017; Quigley, Alvarez Roa, et al., 2020; Quigley, Randall, et al., 2020); *Fugacium* and *Gerakladium* have also been detected at background levels in juveniles (Quigley et al., 2017; Quigley, Alvarez Roa, et al., 2020; Quigley, Randall, et al., 2020). The four genera used here vary in their thermal tolerance (*Fugacium* > Gerakladium > *Durusdinium* > *Cladocopium*; Chakravarti et al., 2017; Chakravarti & van Oppen, 2018), but whether resilience is also conferred to the host after infection has not been studied previously. Our results demonstrate that, surprisingly, when all four symbionts were offered to larvae in equal proportions, rare heterologous symbionts were as, or more, successful relative to the common, homologous species in infecting and proliferating in the coral host across temperatures.

## 2 METHODS

### 2.1 Larval collection and Symbiodiniaceae culture

**FIGURE 1:**
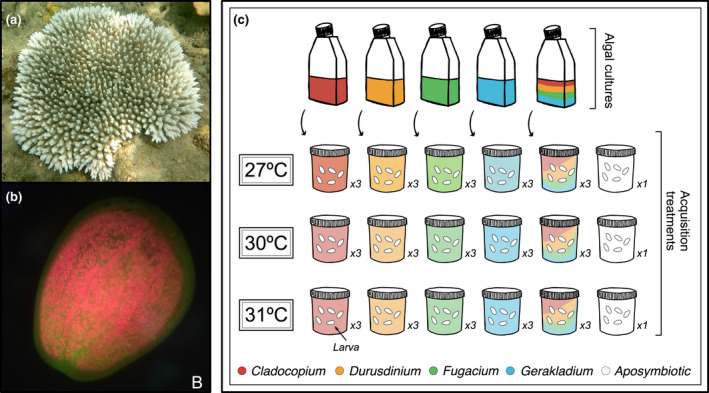
Adult *Acropora tenuis* colony (a) fluorescent microscopy of an *Acropora tenuis* larva (b) (red: Symbiodiniaceae; green: green fluorescent protein). (c) *Acropora tenuis* larvae and algal cultures were acclimated at 27, 30, or 31°C. Symbiont acquisition treatments included no‐choice (*Cladocopium*, *Durusdinium*, *Fugacium,* or *Gerakladium*; 3x per temperature) and four‐way choice (equal mix of all four species). A single jar of aposymbiotic larvae was kept at each temperature. Photo: Hannah Epstein (a), S. Matsuda (b)

Six colonies of *A. tenuis* (Figure 1a) were collected from Falcon Island (18°46′E 146°32′S) in November 2017 (GBRMPA permit # G12/35236.1) 3 days before the full moon and held in tanks at the Australian Institute for Marine Science's (AIMS) National Sea Simulator (SeaSim) research facility (Townsville, Australia). After spawning on the evening of the full moon, gametes were pooled and fertilized as described in Chakravarti et al. (2019). Larvae were reared in ten 420 L flow‐through tanks (0.4 μm filtered sea water, FSW) at ambient temperature (27°C) for 10 weeks until the present experiment commenced (Figure S1). *Acropora tenuis* larvae remained competent (e.g., swimming, not deformed) despite their extended maintenance in tanks. While coral larvae can begin to settle days to weeks post‐spawning, the upper length of duration they are able to remain in the water column is not well known. A study of another acroporid, *Acropora latistella*, and four other species on the Great Barrier Reef found that after an initial drop in survivorship during the first month, there was low mortality until 100 days, with some surviving past 200 days (Graham et al., 2008). In *A*. *tenuis* specifically, Graham et al. (2013) observed a median survivorship of 57 days, at which point larvae were observed to successfully settle. Additionally, Nishikawa et al. (2003) observed that when provided substrate at 60 days post‐spawning, settlement occurred up to 9 days later, highlighting the long duration that *A*. *tenuis* larvae can survive and settle. Lastly, another study used larvae from the same pool as this experiment within the same time frame and observed high larval survival and symbiont acquisition (Buerger et al., 2020).

Four Symbiodiniaceae species in four genera were used in the experiment. These symbiont cultures were originally isolated from four coral species from the Great Barrier Reef and subsequently grown from a monoclonal culture and described in Chakravarti and van Oppen (2018) and Chakravarti et al. (2017) (Table 1). Cultures include *Cladocopium* C1^acro^ (previously referred to as *Cladocopium goreaui*; Beltrán et al., 2021), *Durusdinium* sp., *Fugacium kawagutii*, and *Gerakladium* sp., formerly types C1, D1, F1, and G3, respectively, and hereafter referred to solely by their generic epithets. *In vitro* tolerance for each culture was determined previously for temperatures where cultures experienced positive growth and was as follows: *Cladocopium* 27°C, *Durusdinium* 30°C, *Fugacium* 34°C, and *Gerakladium* 31°C (Chakravarti et al., 2017; Chakravarti & van Oppen, 2018). They were selected for their varying *in vitro* thermal tolerances, with the higher *in vitro* thermal tolerance of the rare species compared to the common and homologous symbiont (*Cladocopium*) being an important selection criterion. Symbiodiniaceae used in this experiment have been maintained in culture (Daigo's IMK, 1% w/v; Nihon Pharmaceutical) at the AIMS Symbiont Culture Facility: Cultures were kept at 27°C in a Steridium E500 growth chamber on a 14:10 h light/dark cycle at 65 ± 10 μmol photons m^−2^ s^−1^ (Sylvania FHO24W/T5/865 fluorescent tubes). The *Cladocopium* C1^acro^ symbiont culture has been previously used successfully in infection studies of larval *A*. *tenuis* (Buerger et al., 2020), and *Acropora spathula* (Quigley, Alvarez Roa, et al., 2020; Quigley, Randall, et al., 2020), juvenile *A*. *tenuis* (Damjanovic et al., 2019), and adults of seven other non‐acroporid species (unpublished; pers. comm. M. J. H. van Oppen).

**TABLE 1:** Symbiodiniaceae cultures used in acquisition experiments from the Australian Institute of Marine Science (Chakravarti & van Oppen, 2018)

Species	Strain ID	ITS2 type	Years in culture	Host species, location	GenBank accession number
*Cladocopium* C1^acro^	SCF 055–01.10	C1	7	*Acropora tenuis*, Nelly Bay, Magnetic Island, Australia	MK027323.1 (Alvarez‐Roa,C. and van Oppen, M.J.)
*Durusdinium* sp.	SCF 086.01	D1	6	*Porites lobata*, Davies Reef, Australia	MH229353.1(Alvarez‐Roa, C. and van Oppen, M.J.)
*Fugacium kawagutii*	SCF 089.01	F1	12	*Pocillopora damicornis*, Heron Island; obtained from the University of Technology Sydney	MK007305.1 (Alvarez‐Roa,C. and van Oppen, M.J.)
*Gerakladium* sp.	SCF 097.01	G3	5	*Diploastrea heliopora*, Davies Reef, Australia	MH229354.1 (Alvarez‐Roa,C. and van Oppen, M.J.)

*Cladocopium* C1^acro^ (Beltrán et al., 2021) has previously been referred to as *Cladocopium goreaui*.

### 2.2 Experimental design

Larvae in this experiment were exposed to Symbiodiniaceae cultures under three temperature treatments (Figure 1c) (in Steridium E500 growth chambers). Prior to the start of larval inoculation experiments, aliquots of both larvae and Symbiodiniaceae were acclimated for 4 days at 27 (ambient), 30 and 31°C under light conditions described above. Approximately 15,000 *A*. *tenuis* larvae were distributed across three 3 L glass jars (*n* = approx. 5000 larvae per jar) in 0.2 µm FSW with one jar incubated at each temperature. After acclimation, larvae in each temperature were transferred into 16 transparent 500 ml plastic jars, which were preconditioned in reverse osmosis water for 72 h prior to the experiment, for inoculation treatments (*n* = 225–250 larvae per jar). Treatments included no‐choice exposures to each of the four species individually and a four‐way choice exposure (mixture of all four species in equal proportions). Each of these five treatments were replicated in three jars at each of the three temperatures, along with one jar per temperature of aposymbiotic controls (*N* = 48 jars). Symbiodiniaceae cell densities in each culture at each temperature treatment were counted on a hemocytometer and the algae were added to larval jars to achieve a final density of 3 × 10^4^ cells/ml. To achieve high initial exposure densities, larvae were inoculated in 100 ml of 0.2 µm FSW for 72 h, thereafter the volume was increased to 400 ml with 50% water changes via pipette every 2–3 days for the duration of the experiment.

### 2.3 Sampling for in hospite Symbiodiniaceae cell density

Symbiodiniaceae cell densities in *A*. *tenuis* larvae were measured on days 3, 7, and 14 during the inoculation experiment; this time frame was chosen because previous *A*. *tenuis* acquisition studies have reported 100% infection rates by days 3–6 (Bay et al., 2011; Yamashita et al., 2014). Cell densities were measured by sampling 20 larvae per jar, however, this sample size was reduced at later time points as numbers declined due to mortality, which is discussed below. Larvae were rinsed twice in 0.2 µm FSW and individually flash frozen in liquid nitrogen at each time point and stored at −80°C for measurement of cell density using Droplet Digital PCR (ddPCR, described below). Additionally, three larvae per jar were sampled and symbiont cells were immediately counted under a fluorescent microscope or by hemocytometer (3 replicates of 3 larvae per jar) (Figure 1b). The fluorescent microscope method was chosen for the initial time point (day 3) due to higher detection capacity at lower densities (larvae were placed under a coverslip with approximately 200 µl of FSW and lightly depressed). Symbionts were visualized by red chlorophyll fluorescence and total cells were counted for each larva. Cell densities were measured using a hemocytometer on days 7 and 14 of the experiment—larvae were pooled from each jar, sonicated in 70 µl of FSW, and counted in two replicate counts on a hemocytometer. Fluorescent microscope and hemocytometer count data were used to analyze acquisition success (binary response) and cell density (cells per larvae) during the experiment. Relative abundance comparisons were analyzed using ddPCR data (described below). Symbiont densities measured by hemocytometer and/or epifluorescence did not differ significantly from those measured by ddPCR (Figure S2; Table S1).

### 2.4 DNA extraction and ddPCR

Genomic DNA was extracted from individual larvae using the protocol outlined in (Wilson et al., 2002). Briefly, samples were placed in an extraction buffer (100 mm Tris pH 9.0, 100 mm EDTA, 100 mm NaCl, 1% SDS, and MilliQ water) with ProteinaseK (20 mg/ml), bead beaten (sterile glass beads, 710–1180 µm, Sigma‐Aldrich G1152; MP Biomedicals FastPrep‐24 4 m/s, 20 s), and incubated (55°C 2 h); 5 m KOAc was added and samples were incubated on ice (30 min) and subsequently centrifuged at 25,000 *g*‐force for 15 min. Incubation was followed by an RNAse A treatment, ethanol cleanup, and resuspension in DNAse‐free water. We used previously developed primers (Table S2). Primers for *Cladocopium* and *Durusdinium* were developed by Cunning and Baker (2012) and amplified the actin gene, and primers targeting ITS2 were used for *Gerakladium* (Meistertzheim et al., 2019) and *Fugacium* (forward: Meistertzheim et al., 2019; reverse: this study, modified from Meistertzheim et al., 2019). ITS2 reverse primer for *Fugacium* were modified using ITS2 sequences (Table S2) from the cultures used in the present study. Target gene copy number (described below) is estimated and accounted for using DNA extracted from counted cells, which normalizes potential variation in copy number among taxa and between different marker genes. All primer pairs were tested on DNA extractions from cultures of all four species using ddPCR to ensure primers were specific to the target species. In these trials there was no evidence of cross‐amplification (Table S3).

Digital droplet polymerase chain reaction (ddPCR) was used to quantify the number of symbiont cells per species per larva. ddPCR partitions reaction mixtures into thousands of nanoliter‐scale water–oil emulsion droplets. PCR amplification occurs within each of the individual droplets and a fluorescence threshold is used to evaluate target amplification in each droplet. Analyzing the fraction of positive and negative droplets on a Poisson distribution is used to estimate the number of gene copies in the original reaction. ddPCR reactions were run on the BioRad QX200 Droplet Digital PCR System using EvaGreen Supermix and following the manufacturer's protocol. Briefly, for each 25 µl reaction, 4 µl DNA template was added to 12.5 µl EvaGreen Supermix, 0.6 µl of each Forward and Reverse Primers (10 µm), and 7.3 µl molecular grade water. For larvae from the four‐way choice experiment, each primer set was run independently (not multiplexed). Twenty microliter of the PCR reaction mix was loaded into a sample well (DG8 Cartridge) with 70 µl of QX200 Droplet Generation Oil for EvaGreen and droplets generated on the QX200 Droplet Generator. PCRs were run on the C1000 Touch Thermal Cycler (95°C 5 min|95°C 30 s, 60°C 60 s (40 cycles)|4°C 5 min, 90°C 5 min) and subsequently read on the QX200 Droplet Reader. Each plate contained negative controls (*n* = 1 per primer set). Fluorescence amplitude minima thresholds for droplets assigned as positive amplification for each primer set were set conservatively within manufacturer guidelines; relative fluorescence intensity thresholds were as follows: *Cladocopium* (13,500), *Durusdinium* (19,000), *Fugacium* (18,000), and *Gerakladium* (16,000). To evaluate false positives, we ran 52 no template control PCR assays (water blanks), ~14 per primer pair. Of these reactions two had one positive droplet and the remaining 50 had zero positive droplets, ascribing an assay false positive rate of 4% at a droplet positivity proportion of 0.01%. We additionally ran 104 control assays on DNA from nominally aposymbiotic larva (19–30 assays per primer pair), which had a droplet positivity proportion <0.03%. Lastly, we evaluated each primer set for specificity by running 6–17 pairwise challenge assays for each combination of primer pair and symbiont (no‐choice larva) for a total of 137 specificity tests: 8 of 14 assays of *Gerakladium* primers × *Fugacium* larva had a droplet positivity proportion of <0.04%, and all other assays maintained a proportion positive <0.02% in less than 1/3 of the tests.

### 2.5 Copy number

To calculate the number of actin or ITS2 gene copies per cell for each species of Symbiodiniaceae, cells from three replicate 300 µl samples of each culture were first counted by hemocytometer, and then DNA was extracted from the remaining 260 µl. Each extraction was serially diluted (1:10, 1:100, 1:1000, 1:10,000), and three replicates of each dilution were analyzed on the ddPCR machine. Copy number for each species was calculated by comparing the number of positive copies (ddPCR) to the number of counted cells (*R* ^2^ > 0.98) and used to convert the number of copies per DNA extraction into the number of Symbiodiniaceae cells per larva.

### 2.6 Data analysis

#### 2.6.1 Larval mortality

All analyses were conducted in R v. 3.6.1 (R Core Team, 2020) and RStudio (1.1.456). Percent survivorship was calculated as the number of larvae alive on days 7 and 14 relative to the total number of larvae in each treatment container at the start of the experiment (day 3); mortality was derived as 1—survivorship. We used binomial mixed effects models to test the effects of treatment, temperature, time, and their interactions on larval mortality using the *lme4* package (Bates et al., 2015). In all mixed effect model analyses in this study, larval jar was included as a random intercept to account for repeated measures over the course of the experiment. Tukey honest significant post hoc tests were used to test for significant pairwise differences. We used Sidak correction to control for familywise error rate conducted pairwise comparisons for each symbiont species using the *emmeans* package (v. 1.4.4; Lenth et al., 2019) in this and all subsequent mixed effect models.

### 2.7 Acquisition success

We collected larval acquisition data (binary, acquisition, or no acquisition) in no‐choice and four‐way choice treatments; in the four‐way choice experiments acquisition success was calculated for each symbiont species. We used binomial mixed effect models to test the effects of choice treatment (no‐choice and four‐way choice treatment), temperature, time, and their interactions on acquisition success. Then, for each symbiont species, we compared acquisition success when offered alone (the no‐choice treatments) and when offered in the presence of other species (four‐way choice).

Treatment groups with *n* < 5, were not included in this, or any subsequent, analysis. These groups included: four‐way choice treatment at 31°C on day 14 and no‐choice *Durusdinium* at 31°C on day 14 across all analyses, as well as cell density in the no‐choice *Fugacium* and *Cladocopium* at 31°C on day 14 (further described below). Due to low larval survivorship on day 14 and at 31°C, models were additionally run on the following comparisons (1) 27 and 30°C, and (2) days 3 and 7, which can be found in the supplemental information.

### 2.8 Symbiont population proliferation

We examined symbiont cell density (cells per larva) for each symbiont species in larvae exposed to no‐choice and four‐way choice experiments that were successfully infected; larvae that were not infected were removed. Cell densities were log_10_ transformed to meet normality assumptions. First, we used a linear mixed effect model to test the effects of treatment (no‐choice and four‐way choice treatment), temperature, time, and their interactions on cell densities. Then, for each species, we compared proliferation (increase over time) when alone (the no‐choice treatment) to proliferation when in competition (four‐way choice).

### 2.9 Interspecies competitive dynamics

The four‐way choice treatment allowed us to examine competitive dynamics between Symbiodiniaceae species. Because ddPCR reactions for each species primer pair were run individually (not multiplexed), there were occasions when reactions for all species in a larva were not successful (i.e., a failed ddPCR, different from an instance of zero symbiont acquisition in a successful ddPCR). Therefore, for all relative abundance models, only larvae where ddPCR reactions were successful for all four species were included. We conducted Kruskal–Wallis non‐parametric tests at each time point to examine the effects of temperature on the number of species that infected larvae. Next, we used linear mixed effects models to test the effects of Symbiodiniaceae species, temperature, time, and their interactions on relative abundance, which was arcsine square root transformed to meet normality assumptions of analyses.

### 2.10 Symbiont community structure

We used multivariate analysis to analyze the effect of temperature treatment and time on symbiont community structure in the four‐way choice treatment. Here, we visualized the variation in symbiont community relative abundance (arcsine square root transformed) among larvae using nonmetric multidimensional scaling (nMDS) based on a Bray–Curtis dissimilarity matrix and evaluation of main effects using a two‐way PERMANOVA (999 permutations).

## 3 RESULTS

### 3.1 Acquisition success: How does temperature and symbiont treatment (no‐choices and the four‐way choice) influence symbiont cell densities within larvae over time?

**FIGURE 2:**
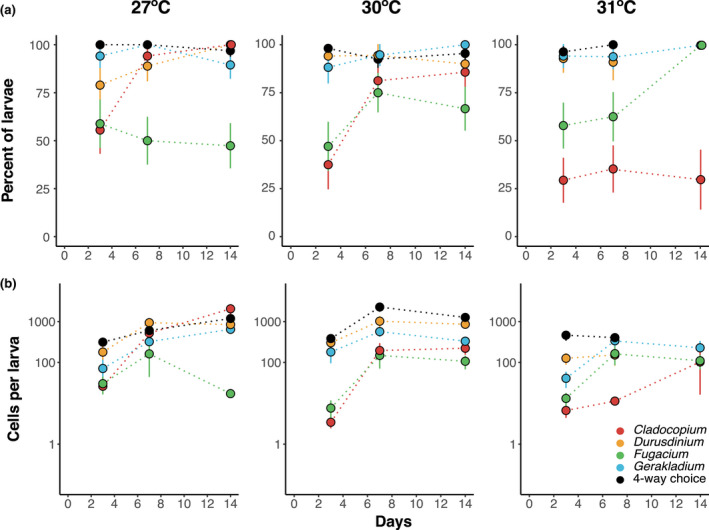
(a) Mean acquisition success (percent of larvae associating with at least one symbiont cell) (±SE) of each species over time (days 3, 7, and 14) at 27, 30, and 31ºC. (b) Mean cell densities (±S.E.) of infected larva, by symbiont infection treatment (no‐choice *Cladocopium*, *Durusdinium*, *Fugacium*, and *Gerakladium*, and the four‐way choice treatment) in *Acropora tenuis* larvae over time at each temperature. Data points on day 14 at 31ºC for no‐choice *Cladocopium*, *Fugacium*, and *Gerakladium* are only included here for visualization purposes

Acquisition success (i.e., the presence of ≥1 cell in a larva) over the course of the experiment differed between symbiont treatments (*p* < .01; Table S4; Figure 2a) but was not influenced by temperature (*p* = .72) or time (*p* = .33) or their interactions. Acquisition success in no‐choice treatments was not uniformly higher in homologous *Cladocopium* than heterologous (*Durusdinium*, *Fugacium,* and *Gerakladium*) symbionts and was high in the four‐way choice experiment across temperature and time (>92%). Across all temperature treatments, larvae exposed to either *Cladocopium* or *Fugacium* symbionts only had the lowest mean acquisition success on day 3 (Figure 2a). However, by day 14, >75% of the larvae at 27 and 30°C were infected in all treatments except the no‐choice *Fugacium*, which was significantly lower with a mean of 47% (27°C) and 67% (30°C).

The only group that showed a difference in acquisition success over time was no‐choice *Cladocopium*. In the no‐choice *Cladocopium* treatment at 27°C, mean acquisition was 56% on day 3, which increased to 100% after 14 days. A similar pattern occurred at 30°C, however, at 31°C, only 30% of larvae established symbioses by day 14.

To understand how infection dynamics change under competition, we compared acquisition success for each species in no‐choice against their performance in the four‐way choice (Figure S3). Acquisition success was not influenced by competition in any temperature treatments or symbiont species (all species *p* > .05; Table S5).

### 3.2 Symbiont proliferation: How does symbiont cell proliferation in larvae differ with temperature?

There were significant effects of symbiont choice treatment (no‐choices or four‐way choice), temperature, time, and their interactions on symbiont cell densities in larvae that had acquired symbionts (*p* ≤ .01; Table 2 and Table S6). Temperature did not affect cell densities within any symbiont choice treatment on day 3 (post hoc, *p* > .05; Figure S4). Between days 3 and 7, mean cell densities increased in all symbiont choice treatments at both ambient and 30°C (Figure 2b). At 27°C, no‐choice *Cladocopium* exhibited the lowest mean cell densities on day 3 (26 cells per larva) but proliferated to the highest mean cell densities of all choice treatments on day 14 (2060 cells per larva). We did not find evidence of a negative effect of 30°C on mean cell density or proliferation in no‐choice *Cladocopium* (Figure S4). However, at 31°C, cell density was significantly reduced and no‐choice *Cladocopium* had the lowest cell densities of all symbiont choice treatments (Figure 2b). No‐choice *Durusdinium* had high cell densities over time and was not negatively impacted by 30°C (post hoc, *p* > .05), however, there were significantly reduced cell densities on day 7 at 31°C (post hoc, *p* = .01). Although there was no difference in cell densities on day 3 in no‐choice *Durusdinium* between temperature treatments (post hoc, *p *> .05), there was significant increase in symbiont cells at 27°C (post hoc, *p* < .01). No‐choice *Fugacium* had lower cell densities than all other choice groups on day 14 at both 27 and 30°C. However, elevated temperature did not negatively affect no‐choice *Fugacium* cell densities at any time point (post hoc, *p* > .05; Figure S4). Interestingly, no‐choice *Gerakladium* was the only treatment to display significant increases in cell density between days 3 and 7 at all temperature treatments (post hoc, *p* < .01; Figure S4).

**TABLE 2:** ANOVA table of mixed effects model analysis of the effect of infection treatment (single‐choices and four‐way choice), temperature, time, and their interactions on larval symbiont cell density (cells per larvae)

Main effects	SS	Num df	Den df	*F*	*p*
Acquisition treatment	**83.53**	**4**	**47.03**	**68.44**	**<0.01**
Day	**67.23**	**2**	**631.14**	**110.17**	**<0.01**
Temp	**6.58**	**2**	**56.06**	**10.79**	**<0.01**
Acquisition treatment:Day	**10.72**	**8**	**631.03**	**4.39**	**<0.01**
Acquisition treatment:Temperature	**11.05**	**8**	**45.39**	**4.53**	**<0.01**
Day:Temperature	**3.40**	**3**	**630.20**	**3.71**	**0.01**
Acquisition treatment:Day:Temperature	**7.88**	**12**	**630.19**	**2.15**	**0.01**

Bold indicates statistical significance.

Abbreviation: SS, sums of squares.

To understand how symbiont choice influenced cell proliferation, we compared cell densities over the course of the experiment for each symbiont species between no‐choice and four‐way choice treatments across the range of temperatures (Table S7; Figure S5). There were higher mean cell densities in no‐choice *Cladocopium* at 27°C on day 7 and day 14 (post hoc, *p *< .01) compared to the four‐way choice. There were also higher cell densities in the no‐choice *Durusdinium* as compared to the four‐way choice on day 7 at 27°C. There was a significant interaction of choice and time (*p* < .01, Table S7) on cell proliferation in *Fugacium* and *Gerakladium* species. *Gerakladium* was the only species that had higher cell densities on day 3 in the four‐way treatment than the no‐choice at all three temperatures (post hoc, *p* < .01), however, there was no difference by day 7 (post hoc, *p* > .05), and no‐choice groups for these species were higher than the four‐way treatment at 27°C by the end of the experiment (647 vs. 299 cells larva^−^; post hoc, *p* = .01).

### 3.3 Relative Abundance of species and mixed assemblage dynamics

**FIGURE 3:**
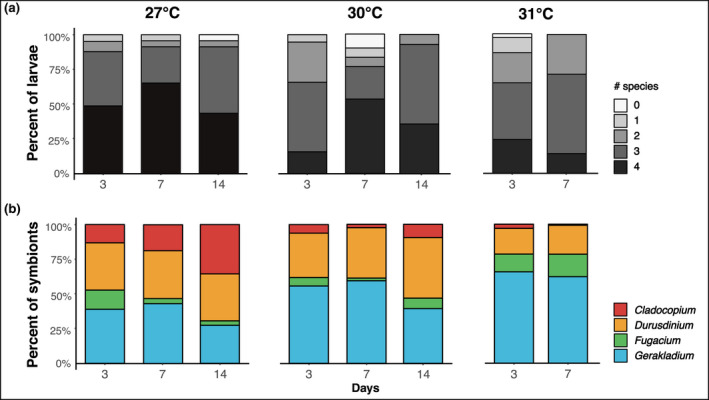
(a) Proportion of four‐way choice larva infected (infection success) by number of symbiont species (zero to four species) across time and temperature treatments. (b) Mean proportion of each symbiont species per larva with successful infection in the four‐way choice treatment over time at each temperature treatment. Number of larvae in each temperature–time point: 27ºC D3: *n* = 41, D7: *n* = 23, D14: *n* = 22; 30ºC D3: *n* = 38, D7: *n* = 27, D14 *n* = 14; 31ºC D3: *n* = 36, D7: *n* = 7

To understand the competitive dynamics of symbiont acquisition in mixed assemblages we examined the symbiont community composition and species abundances in larvae from the four‐way choice treatments. The majority of larvae in the experiment were infected with three (41%) or four species (40%) (Figure S6), and there was a positive correlation between total symbiont cell density and the number of species a larva was associated with (*p* < .01; Table S8, Figure S6). Elevated temperatures significantly reduced the number of species per larva only on day 3 (Kruskal–Wallis, *p* < .01), and reduced the percent of larvae infected with four species at all time points (Figure 3a).

**FIGURE 4:**
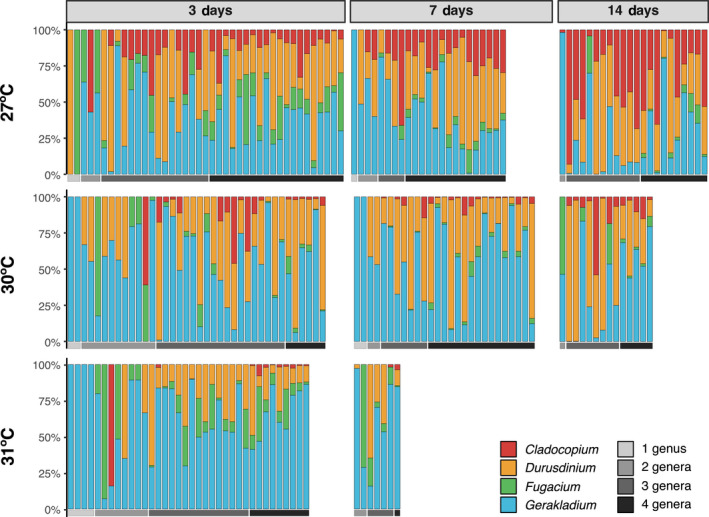
Relative abundance (%) of each symbiont species in an individual larva across time and temperature treatments, ordered by the number of species a larva was infected by (gray scale)

Infectivity and dominance in a larva varied by species. Every symbiotic larva (98.5%) in the four‐way choice experiment (*N* = 208; Table S9) across all temperatures and time points was associated with *Gerakladium*—except for two individuals at 27°C and one individual at 30°C on day 3 (Figure 4). *Durusdinium* infectivity was also high, as it was present in 79% of larvae at 30°C and 72% at 31°C, on day 3. Despite this, at each time point, the number of larvae harboring *Durusdinium* declined with increasing temperatures. *Fugacium* had high infectivity at 27°C (80%), 30°C (53%), and 31°C (78%) on day 3. Despite high infectivity, *Fugacium* was only dominant in a single larva at 30°C and three larvae at 31°C. Elevated temperatures negatively affected the presence and dominance of *Cladocopium*. At 27°C, *Cladocopium* was present in 75% of the three‐species larvae on day 3, despite only being the dominant species in 6%. The infectivity of *Cladocopium* increased over time at this temperature and it was present in 100% of the larvae on day 14 and was the dominant species in 48%. At both elevated temperatures, *Cladocopium* was present in fewer larvae as compared to those at 27°C.

**FIGURE 5:**
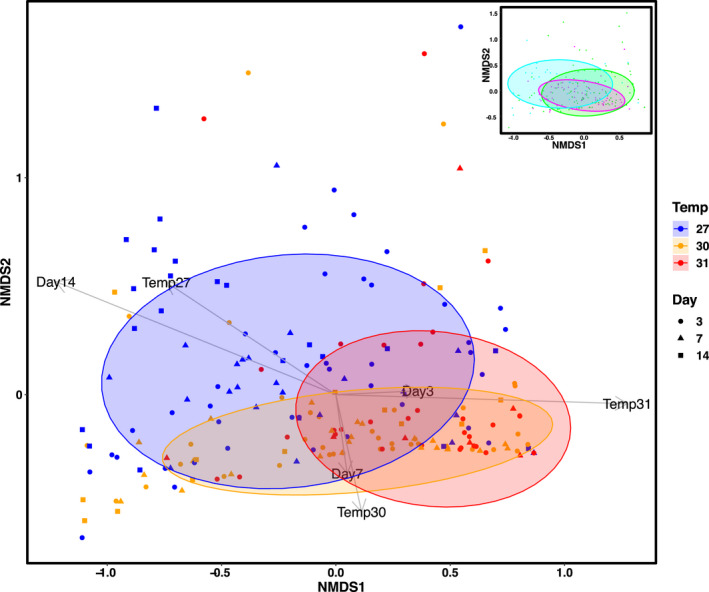
Non‐metric multidimensional scaling (NMDS) plot representing symbiont assemblages in the four‐way choice treatment with 75% ellipses by temperature treatment and ellipses by day in the right top corner insert

Larval symbiont community structure was influenced by both temperature (PERMANOVA, *p* < .01; Table 3) and time (PERMANOVA, *p* < .01; Table 3) (Figure 5) and there was no difference in dispersion between groups (Table S10). There was a species‐specific pattern of relative abundance (*p *< .01), which was further modulated by temperature (*p* < .01) and time (*p* < .01; Table 4 and Table S11). Specifically, at 27°C, *Durusdinium* and *Gerakladium* had the highest mean relative abundances on day 3 (34%: 39%, Figure 3b), which did not change over time (post hoc, *p* > .05). *Cladocopium*, initially at 13%, was the only species to significantly proliferate over time (post hoc, *p* < .01), increasing to 36% by day 14, at which point *Durusdinium*, *Gerakladium*, and *Cladocopium* each represented approximately one third of the relative abundances (Figure 2b). On day 3, the relative abundance of *Fugacium* (14%) was similar to *Cladocopium*, however, over time it was outcompeted (day 14, 3%) by the other three species, and was the only symbiont to significantly decrease in relative abundance over time (27°C, post hoc, *p *= .02).

**TABLE 3:** Two‐way PERMANOVA comparing Symbiodiniaceae diversity between temperature treatments, time, and their interaction

	df	SS	*s*	*F*	*p*
Full model					
Temperature	**2**	**2.69**	**0.14**	**16.86**	**<0.01**
Day	**2**	**0.65**	**0.03**	**4.05**	**<0.01**
Temperature:Day	3	0.10	0.01	0.82	0.55
Residual	200	15.97	0.82		
Total	207	19.51	1		
Day 3					
Temperature	**2**	**1.23**	**0.11**	**6.38**	**<0.01**
Residual	112	9.70	0.89		
Total	114	10.92	1		
Day 7					
Temperature	**2**	**0.65**	**0.17**	**5.62**	**<0.01**
Residual	54	3.17	0.92		
Total	56	3.75	1		
Day 14^a^					
Temperature	1	0.29	0.1	3.08	0.05
Residual	34	3.17	0.9		
Total	35	3.46	1		

Bold indicates statistical significance.

^a^ Day 14 includes 27 and 30°C only due to mortality at 31°C.

**TABLE 4:** ANOVA table of mixed effect model analysis for the four‐way choice larvae of the effect of Symbiodiniaceae genus (*Cladocopium*, *Durusdinium*, *Fugacium,* or *Gerakladium*), time (days), temperature (27, 30, and 31°C), and their interactions on the relative abundances of symbiont cells per larva

Main effects	SS	df Num	df Den	*F*	*p*
Symbiont species	**26.73**	**3**	**800**	**106.65**	**<0.01**
Day	0	2	800	0.02	0.98
Temperature	0.16	2	800	0.93	0.40
Symbiont species:Day	**2.49**	**6**	**800**	**4.96**	**<0.01**
Symbiont species:Temperature	**6.06**	**6**	**800**	**12.08**	**<0.01**
Day:Temperature	0.02	3	800	0.06	0.98
Symbiont species:Day:Temperature	0.88	9	800	1.16	0.31

Bold indicates statistical significance.

Abbreviation: SS, sums of squares.

At elevated temperatures, *Gerakladium's* dominance increased relative to other species. At 30°C, *Gerakladium* outcompeted *Durusdinium* and was the most dominant symbiont on day 3 (56%) and day 7 (59%); however, there was no difference between the two by day 14 (post hoc, *p *> .05). At 31°C, *Gerakladium* was again the most abundant symbiont on day 3 (66%) and day 7 (62%), and there was a greater difference in mean relative abundances between *Gerakladium* and *Durusdinium* at 31°C compared to 30°C. Relative abundance of *Durusdinium* on day 3 did not differ between temperature treatments (post hoc, *p* > .05). *Cladocopium* was less competitive as temperatures increased. *Cladocopium* and *Fugacium* represented low mean relative abundance on day 3 (both 6%); however, unlike *Cladocopium* at 27°C, neither increased over time at 30°C (post hoc, *Cladocopium*: *p *= .99/0.81; *Fugacium*: 0.99/0.99) or 31°C (post hoc, *p* = 1/1).

### 3.4 Larval survival: How did symbiont choice treatment and temperature influence larval survival over time?

**FIGURE 6:**
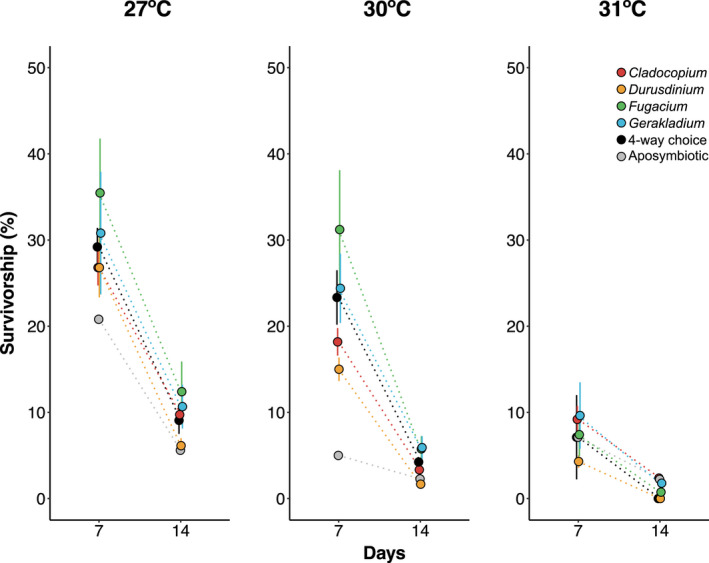
Mean larval survivorship (%) (±SE) in each of the symbiont infection treatments after 7 and 14 days across the experimental temperature (27, 30, and 31ºC) treatments (*n* = 3 jars per temperature treatment). Aposymbiotic controls (gray) included here but not in statistical models due to a single jar per temperature

Larval survivorship decreased over the course of the experiment (*p* < .01) with the lowest survivorship in larvae exposed to the high temperature treatments (*p* < .01; Table S12; Figure 6). Larvae in all symbiont treatments suffered >50% mortality by day 7 of the experiment and by day 14, there was <13% survivorship across all treatments (Figure 6). At 31°C on day 14, there was 100% mortality of larvae exposed to no‐choice *Durusdinium* and the four‐way choice treatments. Survivorship in the aposymbiotic control treatments was lower than or equal to survivorship in the symbiont acquisition treatments (Figure 6, Table S13). The decrease in survivorship across symbiont choice treatments as well as the aposymbiotic control is consistent with other symbiont infection and larval studies that demonstrate high larval mortality (Graham et al., 2008; Randall & Szmant, 2009a; Schnitzler et al., 2012).

Symbiont choice treatments responded differently to elevated temperature (*p* < .01; Table S12). On day 7, larvae in no‐choice *Fugacium* had the highest mean survivorship at 27°C (35%) and 30°C (31%), followed by no‐choice *Gerakladium* (31%, 24%). Larvae in the no‐choice *Fugacium* and *Gerakladium* treatments were the only groups to not experience significantly increased mortality on day 7 at 30°C compared to 27°C (post hoc, *p* = .75, *p* = .16, respectively). Survivorship across all choice treatments dropped significantly between days 7 and 14 (post hoc, *p* < .05), and larvae in all choice treatments had higher survivorship at 27°C than at either of the two elevated temperatures.

Survivorship was not significantly different between the four‐way choice treatment and any of the no‐choice acquisition treatments (post hoc, *p* > .06; Table S12) with the exception of higher survival as compared to *Durusdinium* (post hoc, *p* < .01) and lower survival as compared to *Fugacium* (post hoc, *p* = .01) at 30°C on day 7. At 30°C, however, larvae had higher survivorship in the four‐way choice treatment than those in no‐choice *Durusdinium* treatment (post hoc, *p* < .01), and lower survivorship than the no‐choice *Fugacium* treatment (post hoc, *p* = .01), although these differences were no longer significant by day 14 (post hoc, *p* > .05).

## 4 DISCUSSION

In this study, we examined acquisition dynamics of thermally tolerant heterologous symbionts (*Durusdinium*, *Fugacium,* and *Gerakladium* species) and a homologous thermally sensitive symbiont (*Cladocopium* species) in a no‐choice setting contrasted with acquisition dynamics under four‐way choice in *A. tenuis* larvae. We found that *Gerakladium*, which is normally a background symbiont, achieved similar cell densities to *Cladocopium* and *Durusdinium* at 27°C, and had even higher acquisition and abundance at elevated temperatures (30 and 31°C). These findings show that *A*. *tenuis* larvae can establish symbiosis with *Gerakladium* when symbionts are available at high densities and that *Gerakladium* can infect larvae when *Cladocopium* and *Durusdinium* symbionts are present. We found that the number of algal symbiont species simultaneously acquired by *A*. *tenuis* larvae decreased with temperature, which may be due to the negative effect of elevated temperatures on some of the symbiont species, or possibly, an increased selectivity of *A*. *tenuis* larvae for certain symbiont species at elevated temperatures. Our findings further demonstrate that symbiont identity matters for larval survival under high temperature (Table S12; Figure 6). Here, larvae infected with heterologous *Fugacium* and *Gerakladium* species consistently displayed higher mean survivorship than those infected with *Cladocopium* and *Durusdinium* species, which are common symbionts of *A*. *tenuis* at the larval and juvenile stages (Little et al., 2004; Quigley, Alvarez Roa, et al., 2020; Quigley, Randall, et al., 2020; Yorifuji et al., 2017). Under warming oceans, symbionts normally present at low relative abundances, such as *Gerakladium* and *Fugacium*, may become more abundant symbionts at the larval stage, and if maintained into the juvenile or adult stages may provide benefits. However, this requires experimental testing.

### 4.1 Larval survivorship and symbiont recognition

Summer heat waves occurring during the larval stage will further reduce larval stock and subsequently reduce recruitment (Randall & Szmant, 2009b). Our observations confirm that larvae are negatively impacted by ocean warming; after 2 weeks, elevated temperatures of 30°C moderately reduced survival, with severe mortality at 31°C. These findings suggest that like adult and juvenile *A*. *tenuis* (Shitaoka et al., 2021; Yorifuji et al., 2017), larvae cannot tolerate elevated temperatures beyond 30°C (Shitaoka et al., 2021; Yorifuji et al., 2017). Furthermore, the majority of larvae in the four‐way choice acquisition treatment were infected with a mixed symbiont community (≥3 species per larva) and had a higher mean survivorship after 1 week at 27 and 30°C than those from the no‐choice *Cladocopium* or *Durusdinium* treatments.

Symbiont identity had the most influence on survivorship 1 week after inoculation at 27 and 30°C. Interestingly, larvae inoculated with *Fugacium* under no‐choice had the highest survival, but the lowest cell densities compared to all other treatments. It is possible that the combination of *Fugacium's* high thermal tolerance and low cell densities led to increased larval survivorship, because adult coral colonies with lower symbiont densities have also been observed to have lower bleaching susceptibility (Cunning & Baker, 2012). Other studies have shown increased mortality in symbiotic as compared to aposymbiotic larva at both ambient (Hartmann et al., 2018) and elevated temperatures (Hartmann et al., 2018; Schnitzler et al., 2012), which could be attributed to an increase in oxidative stress (Hartmann et al., 2018), but this may come with other developmental costs (i.e., reduced settlement; Schwarz et al., 1999). Here, we found that survival was not reduced with acquisition of *Cladocopium* and *Durusdinium* symbionts, but there was a trade‐off between acquisition and survival in larvae infected with rare symbionts. Previous work has demonstrated the complexity in the relationship between symbiont acquisition and coral fitness in *A*. *tenuis*; for example, Abrego et al. (2008) found that inoculation with *Cladocopium* resulted in higher thermal tolerance while others (Yorifuji et al., 2017; Yuyama & Higuchi, 2014) found that *Durusdinium* conferred enhanced tolerance. However, there is evidence suggesting that the *A*. *tenuis* population studied in Abrego et al. (2008) from Magnetic Island, GBR, could be a distinct species (Cooke et al., 2020). Therefore, it is possible that associations with other symbiont species or combinations of symbiont species may be more beneficial at the larval life stage.

*Acropora tenuis* larvae exhibit the capacity to host mixed symbiont assemblages (≥2 species) (Cumbo et al., 2013), and we provide evidence that *A*. *tenuis* can form symbioses that can include rare symbiont species in high proportions. Scleractinian corals, including *A*. *tenuis*, exhibit greater flexibility in algal partnerships in early life stages than adults (Abrego et al., 2009a; Gómez‐Cabrera et al., 2008; Coffroth et al., 2001; Little et al., 2004). This flexibility has been linked to host selectivity, availability of a diversity of symbionts, environment factors, and symbiont opportunism during acquisition periods. However, the benefits of hosting different mixed assemblages may be variable (Howe‐Kerr et al., 2020; Putnam et al., 2012). For example, Quigley et al. (2016) found higher mortality in groups of *A*. *tenuis* juveniles with greater symbiont diversity after 25 days, and attributed this to both the low abundance of specific taxa (i.e., A3 and D1) and the negative consequences of random uptake, resulting in suboptimal taxa (i.e., F) that may outcompete higher performers. In contrast, our results in larvae show higher survivorship in the four‐way choice treatment at 14 days, where larvae had high relative abundances of *Fugacium* and *Gerakladium*. This difference could be related to symbiont competition resulting in a trade‐off in juveniles that may not occur in the comparatively shorter larval stage. Although survival benefits were small, these differences were significant and as climate change stressors increase, even marginal gains in survival are important to consider. Here, survivorship after 1 week is influenced the most, and as temperatures and time increase, this effect becomes smaller. However, even the small but significant differences, for example in no‐choice *Fugacium* on day 7 at 30°C, could make the difference for more larvae surviving long enough to settle, which would need to be tested, along with if those symbionts are beneficial at the juvenile stage or not. With the growing trend of annual bleaching on the reef, and the huge impact those events are having on recruitment (Hughes et al., 2019), differences of 5%–10% could make difference or provide a conservation strategy, if also found to be beneficial at later life stages, for those working in management. The winnowing period for *A*. *tenuis* closes on the scale of days (Bay et al., 2011) to years (Abrego et al., 2009a; Gómez‐Cabrera et al., 2008; Rodriguez‐Lanetty et al., 2006). Host requirements may best be met by a mixed assemblage of symbionts during the larval stage that then may be winnowed out as a strategy to maintain the species that best match the environment and host genotype downstream. However, it is important to note that despite evidence for a longer length of the symbiont winnowing period observed in *A*. *tenuis* juveniles, it is possible that the long aposymbiotic larval duration here could influence symbiont specificity, although there are currently no studies testing whether specificity changes over time in larvae.

### 4.2 Background symbionts may be more competitive in warming oceans, if available

*Gerakladium* was a successful and common symbiont partner when available to *A*. *tenuis* larvae in a laboratory setting. *Gerakladium* (subclade G3: MH229354.1) is not a known dominant symbiont in any reef‐building coral, and therefore, less is known about its functional traits *in hospite* (e.g., nutritional translocation, micronutrient requirements). While *Gerakladium* is found predominantly in symbiosis with sponges (Strehlow et al., 2021) and Foraminifera (Pochon & Gates, 2010), and has been observed in soft coral (van Oppen et al., 2005) and black coral (Bo et al., 2011), it has also been detected at background levels in symbiosis with a variety of reef‐building corals (Boulotte et al., 2016; Chakravarti & van Oppen, 2018; LaJeunesse et al., 2010; van Oppen et al., 2005; Quigley et al., 2017; Stat et al., 2013; Thomas et al., 2014). *Gerakladium* is also found in the water column (Cunning et al., 2015; Fujise et al., 2020) and in sediments (Cunning et al., 2015; Fujise et al., 2020) and on macroalgae (Fujise et al., 2020), suggesting it could be an available partner for *A*. *tenuis* larvae on the GBR; however, it is usually present at low abundances (including G3; Quigley, Alvarez Roa, et al., 2020; Quigley, Randall, et al., 2020). However, selectivity is not related to symbiont abundance in the environmental pool (Quigley et al., 2017). A study in the Central West Coast of India, which experienced multiple years of bleaching temperatures, found that despite the symbiont population in the surrounding water containing high abundances of both *Durusdinium* and *Gerakladium*, corals had higher relative abundances of *Durusdinium* (Mote et al., 2021). Despite symbiont assemblages in corals being dominated by *Durusdinium*, *Gerakladium* was also present in higher abundances (10.2 ± 11.7%) than typically found as a background symbiont (Mote et al., 2021). The growing number of observations of *Gerakladium* as a background coral symbiont in a variety of coral species could be a result of increased sampling and molecular methods, or due to an increase in the frequency *Gerakladium* is taken up by corals that might relate to environmental change or stress. Continued monitoring of symbiont assemblages at all life stages, especially on highly impacted reefs, will be necessary to elucidate the degree to which corals are associating with more rare symbiont types.

Like *Gerakladium*, *Fugacium* is not a common dominant coral symbiont. It is predominantly found in symbiosis with Foraminifera (Pochon et al., 2001) and has also been reported at background levels in reef‐building corals (De Palmas et al., 2015; LaJeunesse, 2001; Rodriguez‐Lanetty et al., 2003), and only as the dominant symbiont in *A. japonica*, which is found in temperate environments (De Palmas et al., 2015; LaJeunesse, 2001; Rodriguez‐Lanetty et al., 2003). It has been detected in very low abundance on the GBR (F1; Quigley, Alvarez Roa, et al., 2020; Quigley, Randall, et al., 2020). *Fugacium* is a thermally tolerant symbiont that we now know can also form symbiosis with *A*. *tenuis* larvae. However, *Fugacium* remained at generally low cell densities in larvae at all three temperatures in this study. Chakravarti and van Oppen (2018) tested *Fugacium* culture growth rate and photophysiology and found that cultures exhibited no differences in growth and exhibited the same or higher photosynthetic efficiency at elevated temperature. In this study, no‐choice *Fugacium* had moderate acquisition success at ambient temperature (~50%), with increasing infectivity and competitive capacity at warmer temperatures. The low cell densities could be the result of *A*. *tenuis* not actively attracting *Fugacium*, which could be due to a mismatch in symbiont attraction mechanisms. For example, a recent study by Takeuchi et al. (2021) demonstrated that two different *Fugacium* sp. strains were not attracted to *N*‐acetyl‐d‐glucosamine‐binding lectin on the cell surface of *A*. *tenuis* juveniles, which did attract other species, including *D*. *trenchii*, which may have driven the lower abundances here. Studies have also observed variation in Symbiodiniaceae species attraction towards green fluorescence of *A. tenuis* larvae, for example, with *Durusdinium* (Yamashita et al., 2021). In addition, cell surface recognition mechanisms, such as a glycan–lectin interactions, could limit the uptake of specific symbiont types (Kuniya et al., 2015; Wood‐Charlson et al., 2006) or their persistence in the symbiosis (Parkinson et al., 2018).

There is evidence that smaller symbionts are more readily acquired than larger ones, and therefore size differences between symbiont species here could have contributed to differences in acquisition in this study. While we did not measure symbiont size here, other *Gerakladium* species (*Gerakladium endoclinonum* and *Gerakladium spongiolum*) are, on average, smaller than *Durusdinium*, *Cladocopium,* and *Fugacium* species measured in LaJeunesse et al. (2018); of note, *C*. *goreaui* was larger than the others, while *C*. *thermophilum* was not. Once acquired by the host, symbiont growth rate (population doubles per day) also impacts proliferation within the host. Growth rate for the cultures used here was previously measured in Chakravarti et al. (2017) and Chakravarti and van Oppen (2018). At 27°C in these studies, *Cladocopium* had the highest mean growth rate, however at 31°C, only *Gerakladium* and *Fugacium* exhibited maintained or increased growth rates. *Cladocopium*, on the other hand, exhibited negative growth and *Durusdinium* did not survive (Chakravarti et al., 2017; Chakravarti & van Oppen, 2018). Therefore, enhanced *Gerakladium* and *Fugacium* growth at elevated temperatures could explain their competitive ability in this study under thermal stress and supports our findings that *Cladocopium* exhibited higher proliferation under ambient conditions. This could explain the competitiveness of *Fugacium* and *Gerakladium* at elevated temperatures, and *Cladocopium* at ambient. Algal symbionts with high thermal tolerances could increase host tolerance to warming conditions. Chakravarti and van Oppen (2018) found that cultured wild type *Gerakladium* survived and exhibited population growth at 31°C and *Fugacium* at 34°C, while *Durusdinium* only grew at 30°C. It is possible that *Gerakladium's* thermal tolerance here contributed to the higher survival of larvae with *Gerakladium* at 31°C as compared to *Durusdinium*. During thermal stress, there is a buildup of reactive oxygen species (ROS) in the symbiont, which leak into the host tissues and causes significant damage to the host, leading to bleaching (Lesser, 1997). *Cladocopium* (C1) and *Durusdinium* produced significantly more ROS than *Fugacium* (F2) at ambient (26°C) in an *ex situ* experiment (McGinty et al., 2012). While we did not measure ROS here, there is evidence that thermal tolerance *in vitro* may be transferred to the holobiont (Buerger et al., 2020). Buerger et al. (2020) inoculated *A*. *tenuis* larvae with laboratory heat evolved strains (cultured at 31°C) of *Cladocopium* C1^acro^ (as described in Chakravarti et al., 2017). At 31°C, three of the 10 heat evolved strains conferred higher thermal tolerance to the holobiont (Buerger et al., 2020), and this may be the case for our rare species here. If true, then *A*. *tenuis*’ affinity for thermotolerant symbionts such as *Durusdinium* at the juvenile stage may also translate to symbionts like *Fugacium* or *Gerakladium*, if available, under increasing severe and prolonged temperature stress. However, there may be trade‐offs, as the contribution of background symbionts to the holobiont is still largely unknown, as is the abundance of background symbionts required to produce either a benefit or a disadvantage (Lee et al., 2016). Regardless, there have been observations of novel background symbionts in adult colonies (Rouzé et al., 2017), and as seen here, at the larval stage, the presence of heterologous symbionts does have a positive effect on survivorship. Therefore, further research is warranted to examine nutritional exchange between the coral host and these heterologous symbionts and track implications for host physiology and persistence of these relationships after settlement.

Lastly, natural larval acquisition experiments under ocean warming are occurring on reefs around the world due to global climate change, yet we have not observed large symbiont shifts in adult or juvenile corals on reefs. The number of studies using genetic methods that reveal rare symbionts (e.g., DNA metabarcoding) is still relatively low and recent studies have found that more coral species host diverse assemblages that include rare or background symbionts (Arif et al., 2014; Quigley et al., 2014; Thomas et al., 2014; Varasteh et al., 2021). However, it cannot be discerned whether these rare background symbionts are novel in symbiosis and increasing in presence possibly due to an environmental stressor, or were simply previously undetected. There is evidence that thermally tolerant symbionts are found in symbiosis with corals that live in extreme thermal environments, such as the Red Sea, different from those in surrounding areas (Hume et al., 2016; Ziegler et al., 2017). In these extreme environments, symbiosis with thermotolerant *Cladocopium thermophilum*, only within ~6 ky, has contributed to coral survivorship under increasing temperatures (Hume et al., 2016). While this timeline may not be enough to aid corals under climate change today, it highlights the possibility for coral symbionts to be selected for survival under environmental pressure (Hume et al., 2016). Coral symbiont studies have been predominantly conducted in adults and juveniles and it is difficult to assess symbiont acquisition at the larval stage in the field. Further compounding this is whether symbionts acquired at the larval stage are retained into the juvenile stage, when they would more likely be observed. Given that *A*. *tenuis* may switch from a mixed genera symbiont community at the juvenile stage to a single genus as adults (Abrego et al., 2009a), it is also possible that symbionts acquired at the larval stage differ from those in juveniles. While we may observe selection for thermotolerant homologous symbionts, we cannot rule out the possibility for heterologous symbionts to become more beneficial. *Fugacium* and *Gerakladium* have been found in environmental pools and, critically, our findings show that *A*. *tenuis* larvae *can* acquire these symbionts at high densities even in the presence of the homologous *Cladocopium* and another common heterologous symbiont (*Durusdinium*). This supports our hypothesis that shifts toward rare or background symbionts may occur in coral larvae under future climate change conditions and warrants further study.

### 4.3 Optimizing symbiosis manipulation strategies for assisted evolution

Symbiosis manipulation has been suggested as a possible mechanism of human assisted evolution to aid in coral restoration practices under increasingly warmer conditions (van Oppen et al., 2015). If heterologous symbionts have the capacity to confer higher thermal tolerance than homologous partners, they may be beneficial partners given our rapidly warming climate. Furthermore, inoculating larvae with *Fugacium* and *Gerakladium* may provide phenotypic benefits, even over short periods of time if the symbionts will be replaced by homologous symbionts at a later stage, which has relevance to reef restoration initiatives using sexually produced coral stock. In this context, both *Fugacium* and *Gerakladium* are worth consideration for enhancing climate resilience of coral early life stages in interventions that depend on the rearing of sexually produced coral stock, however, caution should be taken, and further experiments considered before any field implementation occurs. In the present study, there was higher survivorship of larvae infected with *Fugacium* and *Gerakladium* (*Fugacium* > *Gerakladium*) and in larvae offered a four‐way symbiont choice. Our observations at the larval stage point to a new direction in evaluating approaches to symbiont optimization strategies. *Acropora* larvae typically become competent to settle after ~5 days (Graham et al., 2013; Harii et al., 2007). Therefore, survival rates measured at 1 week are ecologically relevant as larvae that have not been swept off the reef or have been transported to another reef on ocean currents will have attached to the substratum and metamorphosed into a coral primary polyp. The impacts on survivorship here were small, but significant. Increasing survivorship under moderately elevated temperatures can enhance the pool of larvae available for settlement and recruitment, which is becoming more important as recruit stocks have shown signs of severe decline in the wake of repeat bleaching events (Hughes et al., 2019). Our results warrant further research to examine whether symbioses with these species can be established at a higher frequency in the laboratory, whether they are temporally stable, and phenotypic implications beyond the larval stage. Considerations should include, first, the impact of mixed assemblages on larval settlement and post‐settlement survival. Second, further research should examine whether winnowing will remove these partners from the assemblage or, alternatively, whether symbiosis with heterologous symbionts, such as *Gerakladium*, could persist into the adult stage. Third, it is important to consider nutritional properties of the symbiont and whether there is a trade‐off between thermal tolerance and capacity to meet the host's energetic needs. Together, these considerations will improve our understanding of the role of currently unusual symbionts in driving host thermal tolerance and fitness. Because both of the strains of *Fugacium* and *Gerakladium* tested here were isolated from cnidarians from the GBR and are present on the GBR (Fujise et al., 2020), this form of assisted evolution would not introduce foreign symbionts to the environment.

## CONFLICT OF INTEREST

The authors declare no conflict of interest.

## AUTHOR CONTRIBUTIONS

SM, LC, MvO, and RG conceived of and designed the study. SM and LC conducted the experiments. SM, AH, CN, and RC analyzed the data. SM and AH prepared the original draft of the manuscript with writing, reviewing, and editing from MvO, LC, CN, and RC.

## Supporting information

Figures S1–S6

Tables S1–S13

## Data Availability

All data and R scripts can be found in Zenodo at doi: https://zenodo.org/badge/latestdoi/227562725

## References

[gcb16057-bib-0001] Abrego, D. , Ulstrup, K. E. , Willis, B. L. , & van Oppen, M. J. H. (2008). Species–specific interactions between algal endosymbionts and coral hosts define their bleaching response to heat and light stress. Proceedings of the Royal Society B: Biological Sciences, 275(1648), 2273–2282. 10.1098/rspb.2008.0180 PMC2603234 18577506

[gcb16057-bib-0002] Abrego, D. , van Oppen, M. J. H. , & Willis, B. L. (2009a). Onset of algal endosymbiont specificity varies among closely related species of *Acropora* corals during early ontogeny. Molecular Ecology, 18(16), 3532–3543. 10.1111/j.1365-294X.2009.04276.x 19627494

[gcb16057-bib-0003] Abrego, D. , van Oppen, M. J. H. , & Willis, B. L. (2009b). Highly infectious symbiont dominates initial uptake in coral juveniles. Molecular Ecology, 18(16), 3518–3531. 10.1111/j.1365-294X.2009.04275.x 19627495

[gcb16057-bib-0004] Aranda, M. , Li, Y. , Liew, Y. J. , Baumgarten, S. , Simakov, O. , Wilson, M. C. , Piel, J. , Ashoor, H. , Bougouffa, S. , Bajic, V. B. , Ryu, T. , Ravasi, T. , Bayer, T. , Micklem, G. , Kim, H. , Bhak, J. , LaJeunesse, T. C. , & Voolstra, C. R. (2016). Genomes of coral dinoflagellate symbionts highlight evolutionary adaptations conducive to a symbiotic lifestyle. Nature Scientific Reports, 1–15. 10.1038/srep39734 PMC5177918 28004835

[gcb16057-bib-0005] Arif, C. , Daniels, C. , Bayer, T. , Banguera‐Hinestroza, E. , Barbrook, A. , Howe, C. J. , LaJeunesse, T. C. , & Voolstra, C. R. (2014). Assessing *Symbiodinium* diversity in scleractinian corals via next‐generation sequencing‐based genotyping of the ITS2 rDNA region. Molecular Ecology, 23(17), 4418–4433. 25052021 10.1111/mec.12869 PMC4285332

[gcb16057-bib-0006] Baird, A. H. , Guest, J. R. , & Willis, B. L. (2009). Systematic and biogeographical patterns in the reproductive biology of Scleractinian corals. Annual Review of Ecology, Evolution, and Systematics, 40(1), 551–571. 10.1146/annurev.ecolsys.110308.120220

[gcb16057-bib-0007] Baird, A. H. , & Marshall, P. A. (2002). Mortality, growth and reproduction in scleractinian corals following bleaching on the Great Barrier Reef. Marine Ecology Progress Series, 237, 133–141. 10.3354/meps237133

[gcb16057-bib-0008] Baker, A. C. (2003). Flexibility and specificity in coral‐algal symbiosis: Diversity, ecology, and biogeography of *Symbiodinium* . Annual Review of Ecology, Evolution, and Systematics, 34(1), 661–689. 10.1146/annurev.ecolsys.34.011802.132417

[gcb16057-bib-0009] Bates, D. , Mächler, M. , Bolker, B. , & Walker, S. (2015). Fitting linear mixed‐effects models using lme4. Journal of Statistical Software, Articles, 67(1), 1–48. 10.18637/jss.v067.i01

[gcb16057-bib-0010] Bay, L. K. , Cumbo, V. R. , Abrego, D. , Kool, J. T. , Ainsworth, T. D. , & Willis, B. L. (2011). Infection dynamics vary between Symbiodinium types and cell surface treatments during establishment of endosymbiosis with coral larvae. Diversity, 3(3), 356–374. 10.3390/d3030356

[gcb16057-bib-0011] Beltrán, V. H. , Puill‐Stephan, E. , Howells, E. , Flores‐Moya, A. , Doblin, M. , Núñez‐Lara, E. , Escamilla, V. , López, T. , & van Oppen, M. J. H. (2021). Physiological diversity among sympatric, conspecific endosymbionts of coral (*Cladocopium* C1^acro^) from the Great Barrier Reef. Coral Reefs, 40(4), 985–997. 10.1007/s00338-021-02092-z

[gcb16057-bib-0012] Berkelmans, R. , & van Oppen, M. J. H. (2006). The role of zooxanthellae in the thermal tolerance of corals: A “nugget of hope” for coral reefs in an era of climate change. Proceedings of the Royal Society B: Biological Sciences, 273(1599), 2305–2312. 10.1098/rspb.2006.3567 PMC1636081 16928632

[gcb16057-bib-0013] Bo, M. , Baker, A. C. , Gaino, E. , Wirshing, H. H. , Scoccia, F. , & Bavestrello, G. (2011). First description of algal mutualistic endosymbiosis in a black coral (Anthozoa: Antipatharia). Marine Ecology Progress Series, 435, 1–11. 10.3354/meps09228

[gcb16057-bib-0014] Boulotte, N. M. , Dalton, S. J. , Carroll, A. G. , Harrison, P. L. , Putnam, H. M. , Peplow, L. M. , & van Oppen, M. J. H. (2016). Exploring the Symbiodinium rare biosphere provides evidence for symbiont switching in reef‐building corals. The ISME Journal, 10(11), 2693–2701. 10.1038/ismej.2016.54 27093048 PMC5113844

[gcb16057-bib-0015] Buerger, P. , Alvarez‐Roa, C. , Coppin, C. W. , Pearce, S. L. , Chakravarti, L. J. , Oakeshott, J. G. , Edwards, O. R. , & van Oppen, M. J. H. (2020). Heat‐evolved microalgal symbionts increase coral bleaching tolerance. Science Advances, 6(20), eaba2498. 10.1126/sciadv.aba2498 32426508 PMC7220355

[gcb16057-bib-0016] Burgess, S. C. , Johnston, E. C. , Wyatt, A. S. J. , Leichter, J. J. , & Edmunds, P. J. (2021). Response diversity in corals: Hidden differences in bleaching mortality among cryptic *Pocillopora* species. Ecology, 102(6), e03324. 10.1002/ecy.3324 33690896 PMC8244046

[gcb16057-bib-0017] Cantin, N. E. , van Oppen, M. J. H. , Willis, B. L. , Mieog, J. C. , & Negri, A. P. (2009). Juvenile corals can acquire more carbon from high‐performance algal symbionts. Coral Reefs, 28(2), 405–414. 10.1007/s00338-009-0478-8

[gcb16057-bib-0018] Chakravarti, L. J. , Beltran, V. H. , & van Oppen, M. J. H. (2017). Rapid thermal adaptation in photosymbionts of reef‐building corals. Global Change Biology, 23(11), 4675–4688. 10.1111/gcb.13702 28447372

[gcb16057-bib-0019] Chakravarti, L. J. , Negri, A. P. , & van Oppen, M. J. H. (2019). Thermal and herbicide tolerances of chromerid algae and their ability to form a symbiosis with corals. Frontiers in Microbiology, 10, 173. 10.3389/fmicb.2019.00173 30809207 PMC6379472

[gcb16057-bib-0020] Chakravarti, L. J. , & van Oppen, M. J. H. (2018). Experimental evolution in coral photosymbionts as a tool to increase thermal tolerance. Frontiers in Marine Science, 5. 10.3389/fmars.2018.00227

[gcb16057-bib-0021] Coffroth, M. A. , Santos, S. R. , & Goulet, T. L. (2001). Early ontogenetic expression of specificity in a cnidarian‐algal symbiosis. Marine Ecology Progress Series, 222, 85–96. 10.3354/meps222085

[gcb16057-bib-0022] Cooke, I. , Ying, H. , Forêt, S. , Bongaerts, P. , Strugnell, J. M. , Simakov, O. , Jia, Z. , Field, M. , Rodriguez‐Lanetty, M. , Bell, S. , Bourne, D. , van Oppen, M. J. H. , Ragan, M. , & Miller, D. J. (2020). Genomic signatures in the coral holobiont reveal host adaptations driven by Holocene climate change and reef specific symbionts. Science Advances, 6(48). 10.1126/sciadv.abc6318 PMC7695477 33246955

[gcb16057-bib-0023] Cumbo, V. R. , Baird, A. H. , & van Oppen, M. J. H. (2013). The promiscuous larvae: Flexibility in the establishment of symbiosis in corals. Coral Reefs, 32(1), 111–120. 10.1007/s00338-012-0951-7

[gcb16057-bib-0024] Cunning, R. , & Baker, A. C. (2012). Excess algal symbionts increase the susceptibility of reef corals to bleaching. Nature Climate Change, 3(3), 259–262. 10.1038/nclimate1711

[gcb16057-bib-0025] Cunning, R. , Silverstein, R. N. , & Baker, A. C. (2018). Symbiont shuffling linked to differential photochemical dynamics of *Symbiodinium* in three Caribbean reef corals. Coral Reefs, 37(1), 145–152. 10.1007/s00338-017-1640-3

[gcb16057-bib-0026] Cunning, R. , Yost, D. M. , Guarinello, M. L. , Putnam, H. M. , & Gates, R. D. (2015). Variability of Symbiodinium communities in waters, sediments, and corals of thermally distinct reef pools in American Samoa. PLoS One, 10(12), e0145099. 10.1371/journal.pone.0145099 26713847 PMC4695085

[gcb16057-bib-0027] Damjanovic, K. , van Oppen, M. J. H. , Menéndez, P. , & Blackall, L. L. (2019). Experimental inoculation of coral recruits with marine bacteria indicates scope for microbiome manipulation in *Acropora tenuis* and *Platygyra daedalea* . Frontiers in Microbiology, 10, 1702. 10.3389/fmicb.2019.01702 31396197 PMC6668565

[gcb16057-bib-0028] De Palmas, S. , Denis, V. , Ribas‐Deulofeu, L. , Loubeyres, M. , Woo, S. , Hwang, S. J. , Song, J. , & Chen, C. A. (2015). *Symbiodinium* spp. associated with high‐latitude scleractinian corals from Jeju Island, South Korea. Coral Reefs, 34(3), 919–925.

[gcb16057-bib-0029] del C. Gómez‐Cabrera, M. , Ortiz, J. C. , Loh, W. K. W. , Ward, S. , & Hoegh‐Guldberg, O. (2008). Acquisition of symbiotic dinoflagellates (*Symbiodinium*) by juveniles of the coral *Acropora longicyathus* . Coral Reefs, 27(1), 219–226. 10.1007/s00338-007-0315-x

[gcb16057-bib-0030] Doropoulos, C. , Ward, S. , Roff, G. , González‐Rivero, M. , & Mumby, P. J. (2015). Linking demographic processes of juvenile corals to benthic recovery trajectories in two common reef habitats. PLoS One, 10(5), e0128535. 10.1371/journal.pone.0128535 26009892 PMC4444195

[gcb16057-bib-0031] Fadlallah, Y. H. (1983). Sexual reproduction, development and larval biology in scleractinian corals. Coral Reefs, 2(3), 129–150. 10.1007/BF00336720

[gcb16057-bib-0032] Fisch, J. , Drury, C. , Towle, E. K. , Winter, R. N. , & Miller, M. W. (2019). Physiological and reproductive repercussions of consecutive summer bleaching events of the threatened Caribbean coral *Orbicella faveolata* . Coral Reefs, 38(4), 863–876. 10.1007/s00338-019-01817-5

[gcb16057-bib-0033] Fujise, L. , Suggett, D. J. , Stat, M. , Kahlke, T. , Bunce, M. , Gardner, S. G. , Goyen, S. , Woodcock, S. , Ralph, P. J. , Seymour, J. , Siboni, N. , & Nitschke, M. R. (2020). Unlocking the phylogenetic diversity, primary habitats, and abundances of free‐living Symbiodiniaceae on a coral reef. Molecular Ecology, 30(1), 343–360. 10.1111/mec.15719 33141992

[gcb16057-bib-0034] Gabay, Y. , Parkinson, J. E. , Wilkinson, S. P. , Weis, V. M. , & Davy, S. K. (2019). Inter‐partner specificity limits the acquisition of thermotolerant symbionts in a model cnidarian‐dinoflagellate symbiosis. The ISME Journal, 13(10), 2489–2499. 10.1038/s41396-019-0429-5 31186513 PMC6776018

[gcb16057-bib-0035] Glynn, P. W. , Mat’e J. L. , Baker, A. C. & Calder’on M. O. (2001). Coral bleaching and mortality in Panama and Ecuador during the 1997–1998 El Niño Southern Oscillation Event: Spatial/temporal patterns and comparisons with the 1982–1983 event. Bulletin of Marine Science, 69(1), 79–109.

[gcb16057-bib-0036] Graham, E. M. , Baird, A. H. , & Connolly, S. R. (2008). Survival dynamics of scleractinian coral larvae and implications for dispersal. Coral Reefs, 27(3), 529–539. 10.1007/s00338-008-0361-z

[gcb16057-bib-0125] Graham, E. M. , Baird, A. H. , Connolly, S. R. , Sewell, M. A. , & Willis, B. L. (2013). Rapid declines in metabolism explain extended coral larval longevity. Coral Reefs, 32(2), 539–549. 10.1007/s00338-012-0999-4

[gcb16057-bib-0037] Harii, S. , Nadaoka, K. , Yamamoto, M. , & Iwao, K. (2007). Temporal changes in settlement, lipid content and lipid composition of larvae of the spawning hermatypic coral Acropora tenuis. Marine Ecology Progress Series, 346, 89–96. 10.3354/meps07114

[gcb16057-bib-0038] Harii, S. , Yamamoto, M. , & Hoegh‐Guldberg, O. (2010). The relative contribution of dinoflagellate photosynthesis and stored lipids to the survivorship of symbiotic larvae of the reef‐building corals. Marine Biology, 157(6), 1215–1224. 10.1007/s00227-010-1401-0

[gcb16057-bib-0039] Hartmann, A. C. , Marhaver, K. L. , Klueter, A. , Lovci, M. T. , Closek, C. J. , Diaz, E. , Chamberland, V. F. , Archer, F. I. , Deheyn, D. D. , Vermeji, M. J. A. , & Medina, M. (2018). Acquisition of obligate mutualist symbionts during the larval stage is not beneficial for a coral host. Molecular Ecology, 28(1), 141–155. 10.1111/mec.14967 30506836

[gcb16057-bib-0124] Hennige, S. J. , Smith, D. J. , Walsh, S.‐J. , McGinley, M. P. , Warner, M. E. , & Suggett, D. J. (2010). Acclimation and adaptation of scleractinian coral communities along environmental gradients within an Indonesian reef system. Journal of Experimental Marine Biology and Ecology, 391(1–2), 143–152. 10.1016/j.jembe.2010.06.019

[gcb16057-bib-0040] Herrera, M. , Klein, S. G. , Campana, S. , Chen, J. E. , Prasanna, A. , Duarte, C. M. , & Aranda, M. (2020). Temperature transcends partner specificity in the symbiosis establishment of a cnidarian. The ISME Journal, 15(1), 141–153. 10.1038/s41396-020-00768-y 32934356 PMC7852570

[gcb16057-bib-0041] Howe‐Kerr, L. I. , Bachelot, B. , Wright, R. M. , Kenkel, C. D. , Bay, L. K. , & Correa, A. M. S. (2020). Symbiont community diversity is more variable in corals that respond poorly to stress. Global Change Biology, 26(4), 2220–2234. 10.1111/gcb.14999 32048447

[gcb16057-bib-0042] Howells, E. J. , Ketchum, R. N. , Bauman, A. G. , Mustafa, Y. , Watkins, K. D. , & Burt, J. A. (2016). Species‐specific trends in the reproductive output of corals across environmental gradients and bleaching histories. Marine Pollution Bulletin, 105(2), 532–539. 10.1016/j.marpolbul.2015.11.034 26608503

[gcb16057-bib-0043] Hughes, T. P. , Kerry, J. T. , Baird, A. H. , Connolly, S. R. , Chase, T. J. , Dietzel, A. , Hill, T. , Hoey, A. S. , Hoogenboom, M. O. , Jacobson, M. , Kerswell, A. , Madin, J. S. , Mieog, A. , Paley, A. , Pratchett, M. S. , Torda, G. , & Woods, R. M. (2019). Global warming impairs stock–recruitment dynamics of corals. Nature, 568(7752), 387–390. 30944475 10.1038/s41586-019-1081-y

[gcb16057-bib-0044] Hughes, T. P. , Kerry, J. T. , Connolly, S. R. , Baird, A. H. , Eakin, C. M. , Heron, S. F. , Hoey, A. S. , Hoogenboom, M. O. , Jacobson, M. , Liu, G. , Pratchett, M. , Skirving, W. , & Torda, G. (2019). Ecological memory modifies the cumulative impact of recurrent climate extremes. Nature Climate Change, 9(1), 40–43. 10.1038/s41558-018-0351-2

[gcb16057-bib-0045] Hume, B. C. C. , Voolstra, C. R. , Arif, C. , D’Angelo, C. , Burt, J. A. , Eyal, G. , Loya, Y. , & Wiedenmann, J. (2016). Ancestral genetic diversity associated with the rapid spread of stress‐tolerant coral symbionts in response to Holocene climate change. Proceedings of the National Academy of Sciences of the USA, 113(16), 4416–4421. 10.1073/pnas.1601910113 27044109 PMC4843444

[gcb16057-bib-0046] Johnston, E. C. , Counsell, C. W. W. , Sale, T. L. , Burgess, S. C. , & Toonen, R. J. (2020). The legacy of stress: Coral bleaching impacts reproduction years later. Functional Ecology, 34(11), 2315–2325. 10.1111/1365-2435.13653

[gcb16057-bib-0047] Jones, A. , & Berkelmans, R. (2010). Potential costs of acclimatization to a warmer climate: Growth of a reef coral with heat tolerant vs. sensitive symbiont types. PLoS One, 5(5). 10.1371/journal.pone.0010437 PMC2862701 20454653

[gcb16057-bib-0048] Jones, A. M. , & Berkelmans, R. (2011). Tradeoffs to thermal acclimation: Energetics and reproduction of a reef coral with heat tolerant *Symbiodinium* type‐D. Journal of Marine Biology, 2011, 1–12. 10.1155/2011/185890

[gcb16057-bib-0127] Kuniya, N. , Jimbo, M. , Tanimoto, F. , Yamashita, H. , Koike, K. , Harii, S. , Nakano, Y. , Iwao, K. , Yasumoto, K. , & Watabe, S. (2015). Possible involvement of Tachylectin‐2‐like lectin from *Acropora tenuis* in the process of *Symbiodinium acquisition* . Fisheries Science, 81(3), 473–483. 10.1007/s12562-015-0862-y

[gcb16057-bib-0049] LaJeunesse, T. C. (2001). Investigating the biodiversity, ecology, and phylogeny of endosymbiotic dinoflagellates in the genus *Symbiodinium* using the its region: In search of a “species” level marker. Journal of Phycology, 37(5), 866–880. 10.1046/j.1529-8817.2001.01031.x

[gcb16057-bib-0050] LaJeunesse, T. C. , Parkinson, J. E. , Gabrielson, P. W. , Jeong, H. J. , Reimer, J. D. , Voolstra, C. R. , & Santos, S. R. (2018). Systematic revision of symbiodiniaceae highlights the antiquity and diversity of coral endosymbionts. Current Biology, 28(16), 2570–2580.e6. 10.1016/j.cub.2018.07.008 30100341

[gcb16057-bib-0051] LaJeunesse, T. C. , Pettay, D. T. , Sampayo, E. M. , Phongsuwan, N. , Brown, B. , Obura, D. O. , Hoegh‐Guldberg, O. , & Fitt, W. K. (2010). Long‐standing environmental conditions, geographic isolation and host–symbiont specificity influence the relative ecological dominance and genetic diversification of coral endosymbionts in the genus Symbiodinium. Journal of Biogeography, 37(5), 785–800.

[gcb16057-bib-0052] LaJeunesse, T. C. , Wiedenmann, J. , Casado‐Amezúa, P. , D’Ambra, I. , Turnham, K. E. , Nitschke, M. R. , Oakley, C. A. , Goffredo, S. , Spano, C. A. , Cubillos, V. M. , Davy, S. K. , & Suggett, D. J. (2021). Revival of Philozoon Geddes for host‐specialized dinoflagellates, ‘zooxanthellae’, in animals from coastal temperate zones of northern and southern hemispheres. European Journal of Phycology, 1–15. 10.1080/09670262.2021.1914863

[gcb16057-bib-0053] Lee, M. J. , Jeong, H. J. , Jang, S. H. , Lee, S. Y. , Kang, N. S. , Lee, K. H. , Kim, H. S. , Wham, D. C. , & LaJeunesse, T. C. (2016). Most low‐abundance “Background” *Symbiodinium* spp. are transitory and have minimal functional significance for symbiotic corals. Microbial Ecology, 71(3), 771–783. 26781946 10.1007/s00248-015-0724-2

[gcb16057-bib-0054] Lenth, R. , Singmann, H. , Love, J. , & Buerkner, P. H. (2019). emmeans: Estimated marginal means, aka least‐squares means. R package v. 1.3. 4.

[gcb16057-bib-0055] Lesser, M. P. (1997). Oxidative stress causes coral bleaching during exposure to elevated temperatures. Coral Reefs, 16(3), 187–192. 10.1007/s003380050073

[gcb16057-bib-0056] Little, A. F. , van Oppen, M. J. H. , & Willis, B. L. (2004). Flexibility in algal endosymbioses shapes growth in reef corals. Science, 304(5676), 1492–1494. 15178799 10.1126/science.1095733

[gcb16057-bib-0057] Manzello, D. P. , Matz, M. V. , Enochs, I. C. , Valentino, L. , Carlton, R. D. , Kolodziej, G. , Serrano, X. , Towle, E. , & Jankulak, M. (2019). Role of host genetics and heat‐tolerant algal symbionts in sustaining populations of the endangered coral *Orbicella faveolata* in the Florida Keys with ocean warming. Global Change Biology, 25(3), 1016–1031. 30552831 10.1111/gcb.14545

[gcb16057-bib-0058] Matsuda, S. , Huffmyer, A. , Lenz, E. A. , Davidson, J. , Hancock, J. , Przybylowski, A. , Innis, T. , Gates, R. , & Barott, K. (2020). Coral bleaching susceptibility is predictive of subsequent mortality within but not between coral species. Frontiers in Ecology and Evolution, 8(178). 10.3389/fevo.2020.00178

[gcb16057-bib-0059] McGinty, E. S. , Pieczonka, J. , & Mydlarz, L. D. (2012). Variations in reactive oxygen release and antioxidant activity in multiple *Symbiodinium* types in response to elevated temperature. Microbial Ecology, 64(4), 1000–1007. 10.1007/s00248-012-0085-z 22767124

[gcb16057-bib-0060] McIlroy, S. E. , Cunning, R. , Baker, A. C. , & Coffroth, M. A. (2019). Competition and succession among coral endosymbionts. Ecology and Evolution, 9(22), 12767–12778. 10.1002/ece3.5749 31788212 PMC6875658

[gcb16057-bib-0061] Meistertzheim, A.‐L. , Pochon, X. , Wood, S. A. , Ghiglione, J.‐F. , & Hédouin, L. (2019). Development of a quantitative PCR–high‐resolution melting assay for absolute measurement of coral‐Symbiodiniaceae associations and its application to investigating variability at three spatial scales. Marine Biology, 166(2), 13. 10.1111/mec.15719

[gcb16057-bib-0062] Mote, S. , Gupta, V. , De, K. , Hussain, A. , More, K. , Nanajkar, M. , & Ingole, B. (2021). Differential Symbiodiniaceae association with coral and coral‐eroding sponge in a bleaching Impacted marginal coral reef environment. Frontiers in Marine Science, 8, 442. 10.3389/fmars.2021.666825

[gcb16057-bib-0063] Muscatine, L. , & Cernichiari, E. (1969). Assimilation of photosynthetic products of zooxanthellae by a reef coral. The Biological Bulletin, 137(3), 506–523. 10.2307/1540172 28368714

[gcb16057-bib-0064] Ng, T. Y. , Chui, A. P. Y. , & Ang, P. (2019). Onset of symbiosis in planula larvae of scleractinian corals. Hydrobiologia, 842(1), 113–126. 10.1007/s10750-019-04030-1

[gcb16057-bib-0065] Nishikawa, A. , Katoh, M. , & Sakai, K. (2003). Larval settlement rates and gene flow of broadcast‐spawning (*Acropora tenuis*) and planula‐brooding (*Stylophora pistillata*) corals. Marine Ecology Progress Series, 256, 87–97. 10.3354/meps256087

[gcb16057-bib-0066] Nitschke, M. R. , Craveiro, S. C. , Brandão, C. , Fidalgo, C. , Serôdio, J. , Calado, A. J. , & Frommlet, J. C. (2020). Description of *Freudenthalidium* gen. nov. and *Halluxium* gen. nov. to formally recognize clades Fr3 and H as genera in thefFamily Symbiodiniaceae (Dinophyceae). Journal of Phycology, 56(4), 923–940. 32267533 10.1111/jpy.12999

[gcb16057-bib-0129] Parkinson, J. E. , Tivey, T. R. , Mandelare, P. E. , Adpressa, D. A. , Loesgen, S. , & Weis, V. M. (2018). Subtle differences in symbiont cell surface glycan profiles do not explain species‐specific colonization rates in a model cnidarian‐algal symbiosis. Frontiers in Microbiology, 9, 1–12. 10.3389/fmicb.2018.00842 29765363 PMC5938612

[gcb16057-bib-0067] Penin, L. , Adjeroud, M. , Schrimm, M. , & Lenihan, H. S. (2007). High spatial variability in coral bleaching around Moorea (French Polynesia): Patterns across locations and water depths. Comptes Rendus Biologies, 330(2), 171–181. 10.1016/j.crvi.2006.12.003 17303544

[gcb16057-bib-0068] Pettay, D. T. , Wham, D. C. , Smith, R. T. , Iglesias‐Prieto, R. , & LaJeunesse, T. C. (2015). Microbial invasion of the Caribbean by an Indo‐Pacific coral zooxanthella. Proceedings of the National Academy of Sciences of the United States of America, 112(24), 7513–7518. 10.1073/pnas.1502283112 26034268 PMC4475936

[gcb16057-bib-0069] Pochon, X. , & Gates, R. D. (2010). A new *Symbiodinium* clade (Dinophyceae) from soritid foraminifera in Hawaiʻi. Molecular Phylogenetics and Evolution, 56(1), 492–497. 10.1016/j.ympev.2010.03.040 20371383

[gcb16057-bib-0070] Pochon, X. , & LaJeunesse, T. C. (2021). *Miliolidium* n. gen, a new Symbiodiniacean genus whose members Associate with soritid foraminifera or are free‐living. The Journal of Eukaryotic Microbiology, e12856. 10.1111/jeu.12856 33966311

[gcb16057-bib-0071] Pochon, X. , Pawlowski, J. , Zaninetti, L. , & Rowan, R. (2001). High genetic diversity and relative specificity among *Symbiodinium*‐like endosymbiotic dinoflagellates in soritid foraminiferans. Marine Biology, 139(6), 1069–1078. 10.1007/s002270100674

[gcb16057-bib-0072] Putnam, H. M. , Stat, M. , Pochon, X. , & Gates, R. D. (2012). Endosymbiotic flexibility associates with environmental sensitivity in scleractinian corals. Proceedings of the Royal Society B: Biological Sciences, 279(1746), 4352–4361. 10.1098/rspb.2012.1454 PMC3479799 22933373

[gcb16057-bib-0073] Qin, Z. , Yu, K. , Chen, B. , Wang, Y. , Liang, J. , Luo, W. , Xu, L. , & Huang, X. (2019). Diversity of Symbiodiniaceae in 15 coral species from the Southern South China Sea: Potential relationship with coral thermal adaptability. Frontiers in Microbiology, 10, 2343. 10.3389/fmicb.2019.02343 31681208 PMC6813740

[gcb16057-bib-0074] Quigley, K. M. , Alvarez, R. C. , Torda, G. , Bourne, D. G. , & Willis, B. L. (2020). Co‐dynamics of Symbiodiniaceae and bacterial populations during the first year of symbiosis with *Acropora tenuis* juveniles. Microbiology Open, 9(2), e959. 31670480 10.1002/mbo3.959 PMC7002099

[gcb16057-bib-0075] Quigley, K. M. , Bay, L. K. , & Willis, B. L. (2017). Temperature and water quality‐related patterns in sediment‐associated *Symbiodinium* communities impact symbiont uptake and fitness of juveniles in the genus Acropora. Frontiers in Marine Science, 4, 401. 10.3389/fmars.2017.00401

[gcb16057-bib-0076] Quigley, K. M. , Davies, S. W. , Kenkel, C. D. , Willis, B. L. , Matz, M. V. , & Bay, L. K. (2014). Deep‐sequencing method for quantifying background abundances of *Symbiodinium* types: Exploring the rare *Symbiodinium* biosphere in reef‐building corals. PLoS One, 9(4). 10.1371/journal.pone.0094297 PMC3984134 24728373

[gcb16057-bib-0077] Quigley, K. M. , Randall, C. J. , van Oppen, M. J. H. , & Bay, L. K. (2020). Assessing the role of historical temperature regime and algal symbionts on the heat tolerance of coral juveniles. Biology Open, 9(1). 10.1242/bio.047316 PMC6994947 31915210

[gcb16057-bib-0078] Quigley, K. M. , Warner, P. A. , Bay, L. K. , & Willis, B. L. (2018). Unexpected mixed‐mode transmission and moderate genetic regulation of *Symbiodinium* communities in a brooding coral. Heredity, 121(6), 524–536. 10.1038/s41437-018-0059-0 29453423 PMC6221883

[gcb16057-bib-0079] Quigley, K. M. , Willis, B. L. , & Bay, L. K. (2016). Maternal effects and symbiodinium community composition drive differential patterns in juvenile survival in the coral *Acropora tenuis* . Royal Society Open Science, 3(10). 10.1098/rsos.160471 PMC5098987 27853562

[gcb16057-bib-0126] R Core Team . (2020). R: A language and environment for statistical computing. R Foundation for Statistical Computing.

[gcb16057-bib-0080] Randall, C. J. , & Szmant, A. M. (2009a). Elevated temperature affects development, survivorship, and settlement of the elkhorn coral, *Acropora palmata* (Lamarck 1816). The Biological Bulletin, 217(3), 269–282. 20040751 10.1086/BBLv217n3p269

[gcb16057-bib-0081] Randall, C. J. , & Szmant, A. M. (2009b). Elevated temperature reduces survivorship and settlement of the larvae of the Caribbean scleractinian coral, *Favia fragum* (Esper). Coral Reefs, 28(2), 537–545.

[gcb16057-bib-0082] Ritson‐Williams, R. , & Gates, R. D. (2020). Coral community resilience to successive years of bleaching in Kane‘ohe Bay, Hawai‘i. Coral Reefs, 10.

[gcb16057-bib-0083] Rodriguez‐Lanetty, M. , Chang, S. J. , & Song, J. I. (2003). Specificity of two temperate dinoflagellate–anthozoan associations from the north‐western Pacific Ocean. Marine Biology, 143(6), 1193–1199. 10.1007/s00227-003-1165-x

[gcb16057-bib-0084] Rodriguez‐Lanetty, M. , Wood‐Charlson, E. M. , Hollingsworth, L. L. , Krupp, D. A. , & Weis, V. M. (2006). Temporal and spatial infection dynamics indicate recognition events in the early hours of a dinoflagellate/coral symbiosis. Marine Biology, 149(4), 713–719. 10.1007/s00227-006-0272-x

[gcb16057-bib-0085] Rouzé, H. , Lecellier, G. J. , Saulnier, D. , Planes, S. , Gueguen, Y. , Wirshing, H. H. , & Berteaux‐Lecellier, V. (2017). An updated assessment of *Symbiodinium* spp. that associate with common scleractinian corals from Moorea (French Polynesia) reveals high diversity among background symbionts and a novel finding of clade B. PeerJ, 5, e2856. 28168100 10.7717/peerj.2856 PMC5289445

[gcb16057-bib-0086] Rowan, R. (2004). Thermal adaptation in reef coral symbionts. Nature, 430(7001), 742. 10.1038/430742a 15306800

[gcb16057-bib-0087] Rowan, R. , Knowlton, N. , Baker, A. , & Jara, J. (1997). Landscape ecology of algal symbionts creates variation in episodes of coral bleaching. Nature, 388(6639), 265–269. 9230434 10.1038/40843

[gcb16057-bib-0088] Schnitzler, C. E. , Hollingsworth, L. L. , Krupp, D. A. , & Weis, V. M. (2012). Elevated temperature impairs onset of symbiosis and reduces survivorship in larvae of the Hawaiian coral, Fungia Scutaria. Marine Biology, 159(3), 633–642. 10.1007/s00227-011-1842-0

[gcb16057-bib-0089] Schwarz, J. A. , Krupp, D. A. , & Weis, M. (1999). Late larval development and onset of symbiosis in the Scleractinian coral *Fungia scutaria* . The Biological Bulletin, 196, 70–79. 25575388 10.2307/1543169

[gcb16057-bib-0090] Shitaoka, R. , Ishibashi, H. , & Takeuchi, I. (2021). Thermal tolerance of the hermatypic coral *Acropora tenuis* elucidated by RGB analysis and expression of heat shock proteins in coral and symbiotic dinoflagellates. Marine Pollution Bulletin, 162, 111812. 10.1016/j.marpolbul.2020.111812 33213856

[gcb16057-bib-0091] Silverstein, R. N. , Correa, A. M. S. , & Baker, A. C. (2012). Specificity is rarely absolute in coral‐algal symbiosis: Implications for coral response to climate change. Proceedings of the Royal Society B, 279(1738), 2609–2618. 22367985 10.1098/rspb.2012.0055 PMC3350700

[gcb16057-bib-0092] Silverstein, R. N. , Cunning, R. , & Baker, A. C. (2015). Change in algal symbiont communities after bleaching, not prior heat exposure, increases heat tolerance of reef corals. Global Change Biology, 21(1), 236–249. 10.1111/gcb.12706 25099991

[gcb16057-bib-0093] Stat, M. , Bird, C. E. , Pochon, X. , Chasqui, L. , Chauka, L. J. , Concepcion, G. T. , Logan, D. , Takabayashi, M. , Toonen, R. , & Gates, R. D. (2011). Variation in *Symbiodinium* ITS2 sequence assemblages among coral colonies. PLoS One, 6(1). 10.1371/journal.pone.0015854 PMC3016399 21246044

[gcb16057-bib-0094] Stat, M. , Loh, W. K. W. , LaJeunesse, T. C. , Hoegh‐Guldberg, O. , & Carter, D. A. (2009). Stability of coral–endosymbiont associations during and after a thermal stress event in the southern Great Barrier Reef. Coral Reefs, 28(3), 709–713. 10.1007/s00338-009-0509-5

[gcb16057-bib-0095] Stat, M. , Morris, E. , & Gates, R. D. (2008). Functional diversity in coral‐dinoflagellate symbiosis. Proceedings of the National Academy of Sciences, 105(27), 9256–9261. 10.1073/pnas.0801328105 PMC2453720 18591663

[gcb16057-bib-0096] Stat, M. , Pochon, X. , Franklin, E. C. , Bruno, J. F. , Casey, K. S. , Selig, E. R. , & Gates, R. D. (2013). The distribution of the thermally tolerant symbiont lineage (*Symbiodinium* clade D) in corals from Hawaii: Correlations with host and the history of ocean thermal stress. Ecology and Evolution, 3(5), 1317–1329. 10.1002/ece3.556 23762518 PMC3678486

[gcb16057-bib-0097] Strehlow, B. W. , Pineda, M.‐C. , Kenkel, C. D. , Laffy, P. , Duckworth, A. , Renton, M. , Clode, P. L. , & Webster, N. S. (2021). Novel reference transcriptomes for the sponges *Carteriospongia foliascens* and *Cliona orientalis* and associated algal symbiont *Gerakladium endoclionum* . Coral Reefs, 40(1), 9–13. 10.1007/s00338-020-02028-z

[gcb16057-bib-0098] Suzuki, G. , Yamashita, H. , Kai, S. , Hayashibara, T. , Suzuki, K. , Iehisa, Y. , Okada, W. , Ando, W. , & Komori, T. (2013). Early uptake of specific symbionts enhances the post‐settlement survival of Acropora corals. Marine Ecology Progress Series, 494, 149–158. 10.3354/meps10548

[gcb16057-bib-0099] Takeuchi, R. , Jimbo, M. , Tanimoto, F. , Iijima, M. , Yamashita, H. , Suzuki, G. , Harii, S. , Nakano, Y. , Yasumoto, K. , & Watabe, S. (2021). N‐Acetyl‐D‐Glucosamine‐Binding Lectin in *Acropora tenuis* attracts specific Symbiodiniaceae cell culture strains. Marine Drugs, 19(146). 10.3390/CS-161 PMC8002028 33799701

[gcb16057-bib-0100] Teschima, M. M. , Garrido, A. , Paris, A. , Nunes, F. L. D. , & Zilberberg, C. (2019). Biogeography of the endosymbiotic dinoflagellates (Symbiodiniaceae) community associated with the brooding coral *Favia gravida* in the Atlantic Ocean. PLoS One, 14(4), e0215167. 30849101 10.1371/journal.pone.0213519 PMC6407780

[gcb16057-bib-0101] Thiault, L. , Curnock, M. I. , Gurney, G. G. , Heron, S. F. , Marshall, N. A. , Bohensky, E. , Nakamura, N. , Pert, P. L. , & Claudet, J. (2020). Convergence of stakeholders’ environmental threat perceptions following mass coral bleaching of the Great Barrier Reef. Conservation Biology, 35(2), 598–609. 10.1111/cobi.13591 32681546

[gcb16057-bib-0102] Thomas, L. , Kendrick, G. A. , Kennington, W. J. , Richards, Z. T. , & Stat, M. (2014). Exploring *Symbiodinium* diversity and host specificity in Acropora corals from geographical extremes of Western Australia with 454 amplicon pyrosequencing. Molecular Ecology, 23(12), 3113–3126. 24845644 10.1111/mec.12801

[gcb16057-bib-0103] van Oppen, M. J. H. , Mahiny, A. J. , & Done, T. J. (2005). Geographic distribution of zooxanthella types in three coral species on the Great Barrier Reef sampled after the 2002 bleaching event. Coral Reefs, 24(3), 482–487. 10.1007/s00338-005-0487-1

[gcb16057-bib-0104] van Oppen, M. J. H. , McDonald, B. J. , Willis, B. , & Miller, D. J. (2001). The evolutionary history of the coral genus Acropora (Scleractinia, Cnidaria) based on a mitochondrial and a nuclear marker: Reticulation, incomplete lineage sorting, or morphological convergence? Molecular Biology and Evolution, 18(7), 1315–1329. 11420370 10.1093/oxfordjournals.molbev.a003916

[gcb16057-bib-0105] van Oppen, M. J. H. , Mieog, J. C. , Sánchez, C. A. , & Fabricius, K. E. (2005). Diversity of algal endosymbionts (zooxanthellae) in octocorals: The roles of geography and host relationships. Molecular Ecology, 14(8), 2403–2417. 10.1111/j.1365-294X.2005.02545.x 15969723

[gcb16057-bib-0106] van Oppen, M. J. H. , Oliver, J. K. , Putnam, H. M. , & Gates, R. D. (2015). Building coral reef resilience through assisted evolution. Proceedings of the National Academy of Sciences of the United States of America, 112(8), 2307–2313. 10.1073/pnas.1422301112 25646461 PMC4345611

[gcb16057-bib-0107] Varasteh, T. , Salazar, V. , Tschoeke, D. , Francini‐Filho, R. B. , Swings, J. , Garcia, G. , Thompson, C. C. , & Thompson, F. L. (2021). *Breviolum* and *Cladocopium* are dominant among Symbiodiniaceae of the coral holobiont *Madracis decactis* . Microbial Ecology. 10.1007/s00248-021-01868-8 34561754

[gcb16057-bib-0108] Wall, C. B. , Kaluhiokalani, M. , Popp, B. N. , Donahue, M. J. , & Gates, R. D. (2020). Divergent symbiont communities determine the physiology and nutrition of a reef coral across a light‐availability gradient. The ISME Journal, 14(4), 945–958. 10.1038/s41396-019-0570-1 31900444 PMC7082336

[gcb16057-bib-0109] Ward, S. , Harrison, P. , & Hoegh‐Guldberg, O. (2002). Coral bleaching reduces reproduction of scleractinian corals and increases susceptibility to future stress. Proceedings of the Ninth International Coral Reef Symposium, Bali, 23‐27 October 2000, Vol. 2, pp. 1123–1128.

[gcb16057-bib-0110] Weis, V. M. , Reynolds, W. S. , deBoer, M. D. , & Krupp, D. A. (2001). Host‐symbiont specificity during onset of symbiosis between the dinoflagellates *Symbiodinium* spp. and planula larvae of the scleractinian coral *Fungia scutaria* . Coral Reefs, 20(3), 301–308.

[gcb16057-bib-0111] Wilson, K. , Li, Y. , Whan, V. , Lehnert, S. , Byrne, K. , Moore, S. , Pongsomboon, S. , Tassanakajon, A. , Rosenberg, G. , Ballment, E. , Fayazi, Z. , Swan, J. , Kenway, M. , & Benzie, J. (2002). Genetic mapping of the black tiger shrimp Penaeus monodon with amplified fragment length polymorphism. Aquaculture, 204(3–4), 297–309. 10.1016/S0044-8486(01)00842-0

[gcb16057-bib-0128] Wood‐Charlson, E. M. , Hollingsworth, L. L. , Krupp, D. A. , & Weis, V. M. (2006). Lectin/glycan interactions play a role in recognition in a coral/dinoflagellate symbiosis. Cellular Microbiology, 8(12), 1985–1993. 10.1111/j.1462-5822.2006.00765.x 16879456

[gcb16057-bib-0112] Yakovleva, I. , Baird, A. , Yamamoto, H. H. , Bhagooli, R. , Nonaka, M. , & Hidaka, M. (2009). Algal symbionts increase oxidative damage and death in coral larvae at high temperatures. Marine Ecology Progress Series, 378, 105–112. 10.3354/meps07857

[gcb16057-bib-0113] Yamashita, H. , Koike, K. , Shinzato, C. , Jimbo, M. , & Suzuki, G. (2021). Can *Acropora tenuis* larvae attract native Symbiodiniaceae cells by green fluorescence at the initial establishment of symbiosis? PLoS One, 16(6), e0252514. 10.1371/journal.pone.0252514 34061893 PMC8168901

[gcb16057-bib-0114] Yamashita, H. , Suzuki, G. , Kai, S. , Hayashibara, T. , & Koike, K. (2014). Establishment of coral–algal symbiosis requires attraction and selection. PLoS One, 9(5), e97003. 10.1371/journal.pone.0097003 24824794 PMC4019531

[gcb16057-bib-0115] Yorifuji, M. , Harii, S. , Nakamura, R. , & Fudo, M. (2017). Shift of symbiont communities in *Acropora tenuis* juveniles under heat stress. PeerJ, 5, e4055. 10.7717/peerj.4055 29255647 PMC5732543

[gcb16057-bib-0116] Yuyama, I. , & Higuchi, T. (2014). Comparing the effects of symbiotic algae (Symbiodinium) clades C1 and D on early growth stages of *Acropora tenuis* . PLoS One, 9(6), 1–7. 10.1371/journal.pone.0098999 PMC4051649 24914677

[gcb16057-bib-0117] Ziegler, M. , Arif, C. , Burt, J. A. , Dobretsov, S. , Roder, C. , LaJeunesse, T. C. , & Voolstra, C. R. (2017). Biogeography and molecular diversity of coral symbionts in the genus *Symbiodinium* around the Arabian Peninsula. Journal of Biogeography, 44(3), 674–686. 28286360 10.1111/jbi.12913 PMC5324606

