# Supplementary material for: Temperature‐mediated acquisition of rare heterologous symbionts promotes survival of coral larvae under ocean warming
Source: Glob Chang Biol. 2022 Jan 5;28(6):2006–25. doi: 10.1111/gcb.16057 (PMC9303745; doi:10.1111/gcb.16057)
Supplement: Supplementary file 1 — Figures S1–S6 [file GCB-28-2006-s002.pdf]

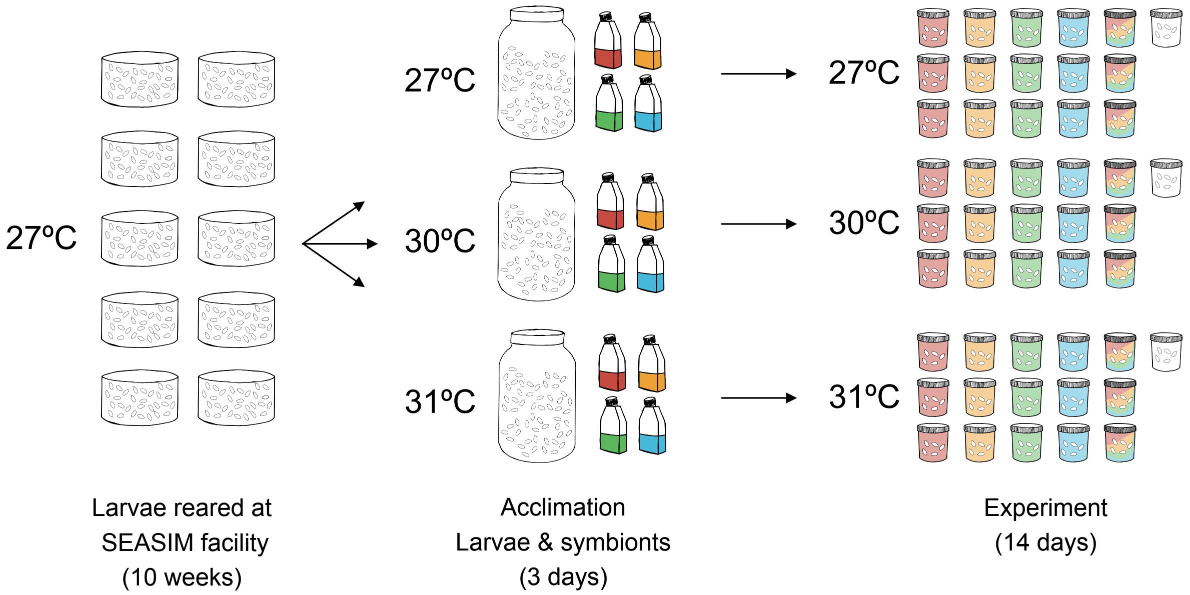

Figure S1: Larvae were reared at the Australian Institute for Marine Science's National Sea Simulator (SeaSim) research facility and held in 420 L flow-through tanks (0.4 µm filtered sea water (FSW)) for 10 weeks at 27ºC. A subset of larvae (~5,000 per temperature treatment) were then acclimated at either 27, 30 or 31ºC (0.2 µm FSW) for four days (symbiont culture were also acclimated to these temperatures separately from the larvae). Larvae were dived into 500 mL jars (~225-250 larvae per jar) and inoculated with the no-choice or 4-way choice symbiont treatments.

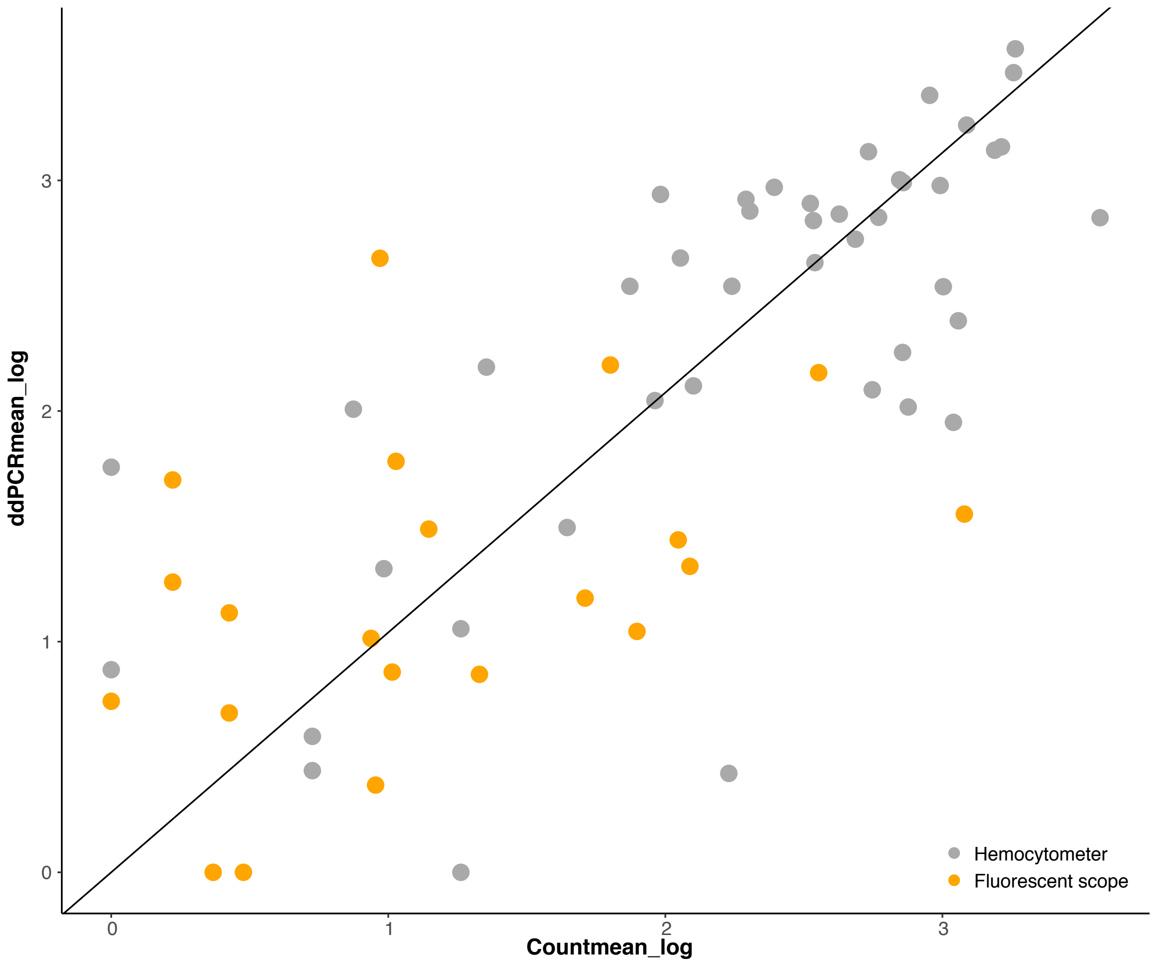

Figure S2: Model2 linear regression of ddPCR by hemocytometer, epifluorescence and combined cell counts. The 95% confidence intervals of all three slopes are not significantly different from a 1:1 relationship (Table S1). ddPCR, hemocytometer, and fluorescent scope counts are log transformed.

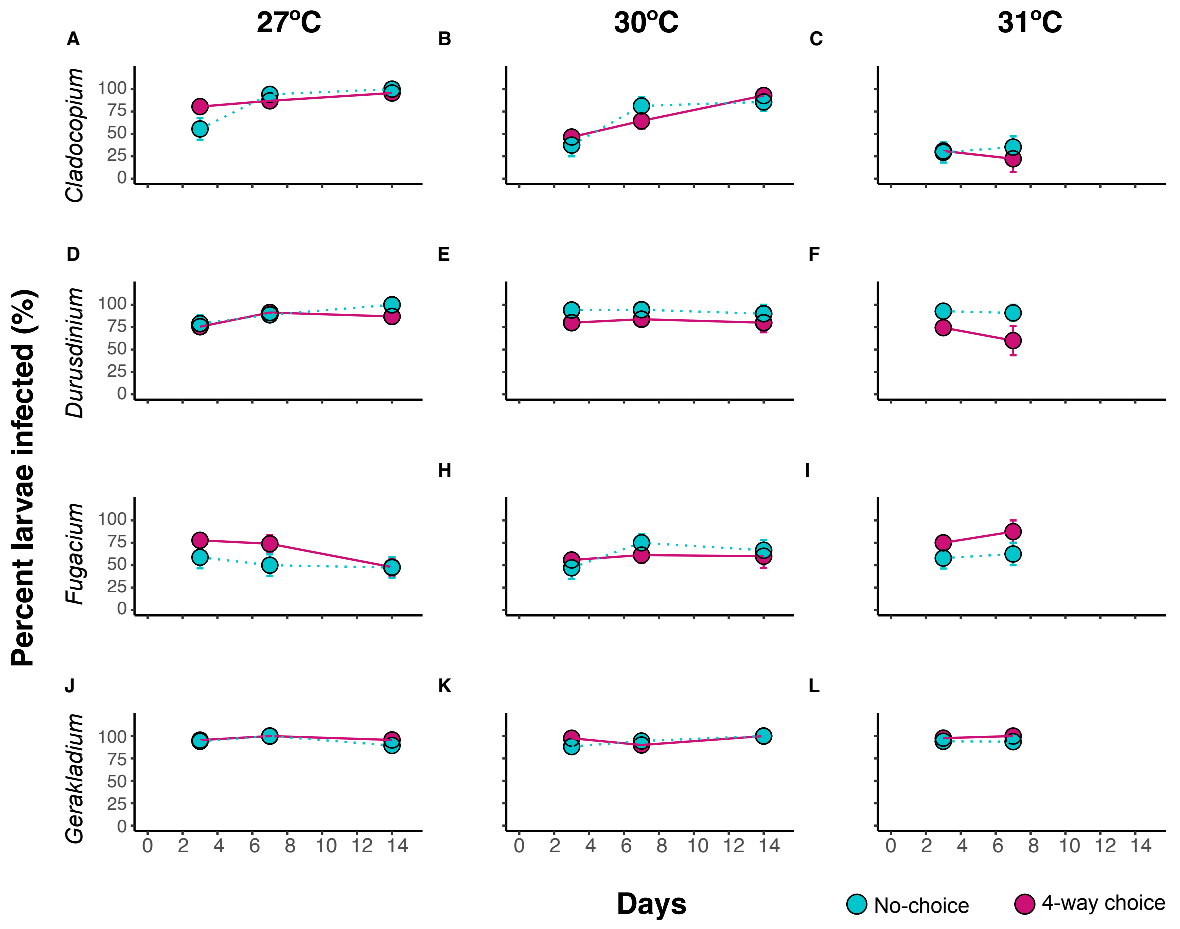

Figure S3: Percent of larvae infected (infection success) (± s.e.) of each species in single-choice (pink) or 4-way choice (blue) over time (days 3, 7 and 14) at 27, 30, and 31ºC. Data points only included if n > 5 for each treatment pair. There are no significant differences between treatments.

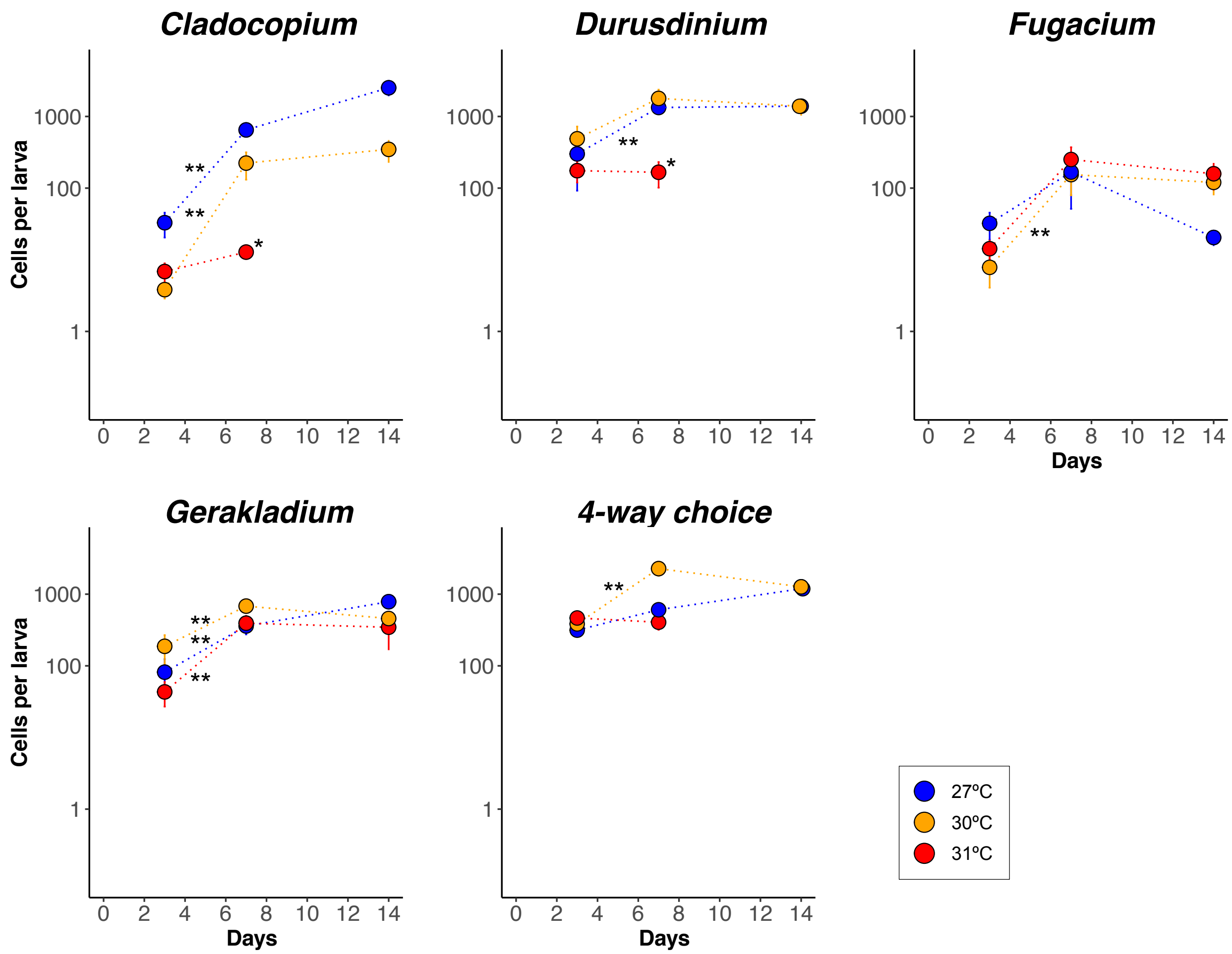

Figure S4. Mean cell counts for the single-choices and the 4-way choice treatments over time and by temperature (27ºC = blue, 30ºC = yellow, 31ºC = red) by species. * = significant differences between temperatures at a time point, ** = significant difference between time points at a temperature.

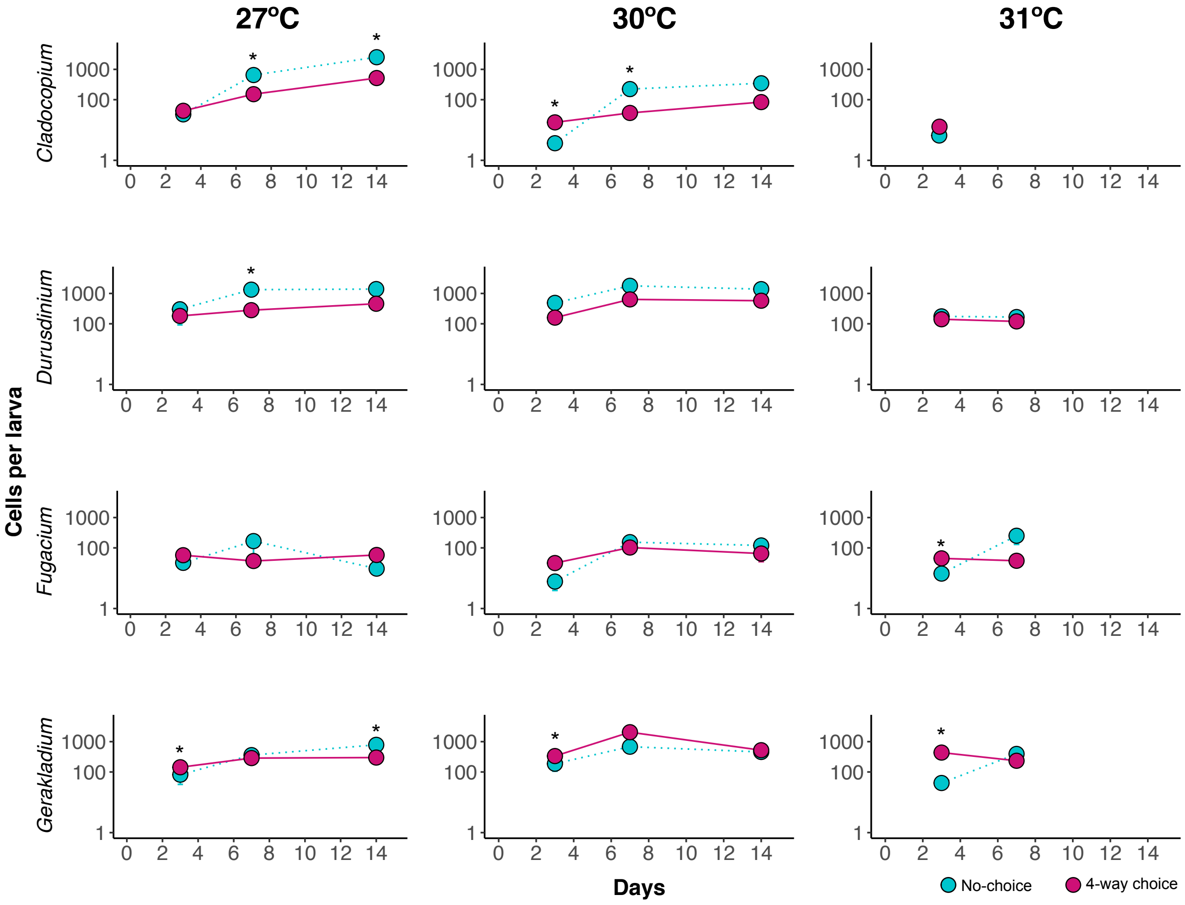

Figure S5: Mean cell densities (± s.e.) of each genus in monoculture infection (blue) and under competition in the polyculture (pink) across time at 27, 30, and 31ºC. * = significant difference at a time point, ** = significant difference between time points. Data points only included if n > 5 for both treatments in a pair at a time point.

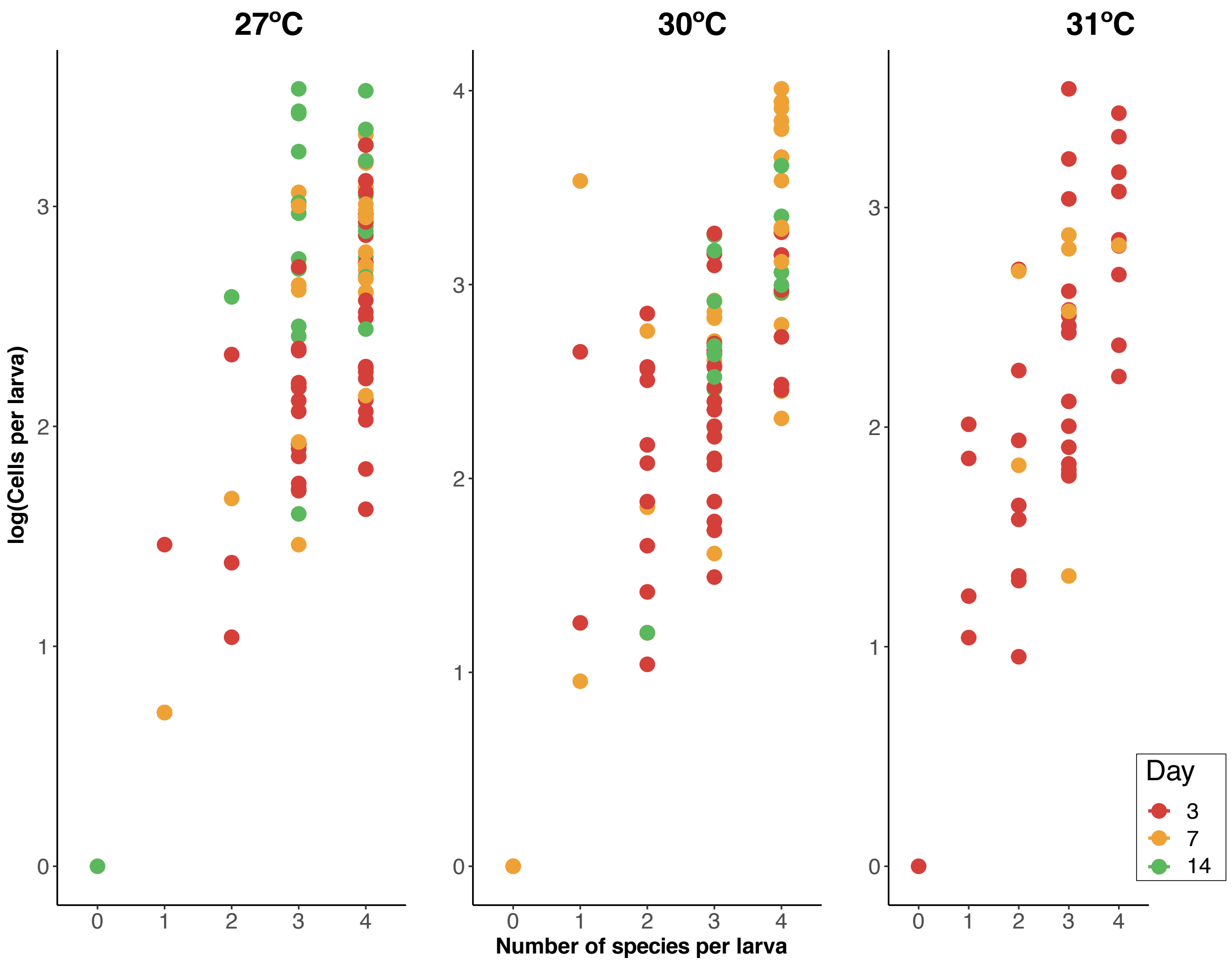

Figure S6. Cells per larva (log transformed) by number of species a larva was infected with, across time at 27, 30 and 31ºC. At each temperature, cells per larva increase with the number of species per larva.
