# Supplementary material for: Temperature‐mediated acquisition of rare heterologous symbionts promotes survival of coral larvae under ocean warming
Source: Glob Chang Biol. 2022 Jan 5;28(6):2006–25. doi: 10.1111/gcb.16057 (PMC9303745; doi:10.1111/gcb.16057)
Supplement: Supplementary file 2 — Tables S1–S13 [file GCB-28-2006-s001.pdf]

Supplemental Tables

Table S1. Model2 linear regression of ddPCR by hemocytometer, epifluorescence and combined cell counts. The 95% confidence intervals of all three slopes are not significantly different from a 1:1 relationship. Additionally, there is no significant interaction (p = 0.07) with count method on ddPCR counts (lm(ddPCR~Counts+Counts:Count.type), which is apparent in that the 95% confidence intervals of each of the three slope coefficients overlap.

Main Effects	Intercept	Slope	2.5%-Slope	97.5%-Slope
Scope+hemocytometer	0.14	0.96	0.76	1.20
Scope	0.47	0.63	0.09	1.66
Hemocytometer	0.10	0.99	0.73	1.34

Table S2. Primers used for ddPCR for each Symbidiniaceae species. BP = amplicon size.

**ITS2 type**	**gene**	**Primer pair**	**Sequence**	**BP**	**Citation**
C1	actin	Cact_F	CCA GGT GCG ATG TCG ATA TTC	96	(Cunning and Baker 2012)
		Cact_R	TGG TCA TTC GCT CAC CAA TG		(Cunning and Baker 2012)
D1	actin	Dact_F	GGC ATG GGG TAA GCA CTT CTT	106	(Cunning and Baker 2012)
		Dact_R	GAT CCT TGA ACT AGC CTT GGA AAC		(Cunning and Baker 2012)
F1	its2	F1its2_For	GCC CCT GTG AGC CAT TGA	91	(Meistertzheim et al. 2019)
		F1its2_Rev.2	AAG GTG GAA TCT TGA ATA GCA TCG		This study (modified Meistertzheim et al. 2019)
G3	its2	Gits2_F	GCC TCG GCG TGT TGT TG	72	(Meistertzheim et al. 2019)
		Gits2_R	AGC ACG TGC ATG CTT GCA		(Meistertzheim et al. 2019)

Table S3: Percent of droplets called as positive/negative for each species' primer pair when tested on DNA extracted from cultures of the three other species using ddPCR.

	DNA extracted from cultures			
Primers	*Cladocopium*	*Durusdinium*	*Fugacium*	*Gerakladium*
Cladocopium		0.000012026	0.000027955	0.000006690
Durusdinium	0.000007106		0.000000000	0.000006693
Fugacium	0.000009309	0.000082642		0.000009968
Gerakladium	0.000009969	0.000054263	0.000100063	

Table S4. Binomial mixed effects model table of the effect of infection treatment (four no-choice and the 4-way choice), temperature (27, 30, and 31ºC), time (days) and their interactions on the number of larvae that established symbioses. Bold indicates statistical significance ($p < 0.05$).

			Full	27C & 30C	Day 3 & 7
Main effects	Chisq	Df	P	P	P
**Infection Treatment**	**92.94**	**13**	**<0.01**	**<0.01**	**<0.01**
Temp	3.68	6	0.72	0.69	0.70
Day	11.36	10	0.33	0.17	0.12
Symbiont:Temp	11.75	8	0.16	0.39	0.26
Symbiont:Day	9.85	9	0.36	0.19	0.18
Temp:Day	3.68	3	0.30	0.61	0.34
Symbiont:Temp:Day	3.74	12	0.99	0.97	0.88

Table S5. Binomial mixed effects model results by genus testing the effect of competition (no-choices vs 4-way choice by species), time (days), temperature, and their interactions on infection success under temperature treatment.

	Main effects	Chisq	Df	Pr(>Chisq)
Cladocopium	**Temperature**	**36.56**	**2**	**<0.01**
	Infection Type	0.30	1	0.58
	**Day**	**16.52**	**2**	**<0.01**
	Temperature:Infection Type	1.21	2	0.55
	Temperature:Day	5.07	3	0.17
	Infection Type:Day	4.27	2	0.12
	Temperature:Infection Type:Day	0.58	3	0.90
Durusdinium	Temperature	1.70	2	0.43
	Infection type	3.59	1	0.06
	Day	0.52	1	0.47
	Temperature:Infection Type	3.27	2	0.20
	Temperature:Day	3.18	2	0.20
	Infection Type:Day	0.04	1	0.84
	Temperature:Infection Type:Day	0.18	2	0.92
Fugacium	Temperature	1.51	2	0.47
	Infection Type	2.47	1	0.12
	Day	2.42	2	0.30
	Temperature:Infection Type	2.80	2	0.25
	Temperature:Day	4.99	3	0.17
	Infection Type:Day	1.33	2	0.51
	Temperature:Infection Type:Day	1.84	3	0.61
Gerakladium	Temperature	0.34	3	0.95
	Infection Type	2.48	2	0.29
	Day	2.11	4	0.72
	Temperature:Infection Type	0.62	2	0.73
	Temperature:Day	0.21	4	1.00
	Infection Type:Day	1.91	2	0.38
	Temperature:Infection Type:Day	0.00	3	1.00

Table S6. (*Tab. 2 with subsets added*) ANOVA table of mixed effects model analysis of the effect of infection treatment (no-choices and 4-way choice), temperature), time, and their interactions on larval symbiont cell density (cells/larvae). Subsetted data includes 1) 27 and 30C only, and 2) Days 3 and 7 only. SS = sums of squares.

					Full	27C and 30C	D3 and D7
Main effects	SS	Num df	Den df	F	P	P	P
**Infection Treatment**	**83.53**	**4**	**47.03**	**68.44**	**<0.01**	**<0.01**	**<0.01**
**Day**	**67.23**	**2**	**631.14**	**110.17**	**<0.01**	**<0.01**	**<0.01**
**Temp**	**6.58**	**2**	**56.06**	**10.79**	**<0.01**	0.15	**<0.01**
**Infection Treatment:Day**	**10.72**	**8**	**631.03**	**4.39**	**<0.01**	**<0.01**	**<0.01**
**Infection Treatment:Temperature**	**11.05**	**8**	**45.39**	**4.53**	**<0.01**	**<0.01**	**<0.01**
**Day:Temperature**	**3.40**	**3**	**630.20**	**3.71**	**0.01**	0.83	**0.01**
**Symbiont:Day:Temperature**	**7.88**	**12**	**630.19**	**2.15**	**0.01**	**0.03**	0.07

Table S7. ANOVA linear mixed effects model summary table for each symbiont genus testing the effect of competition (alone (no-choice) or competition (4-way choice)), day, temperature, and their interactions on cell densities. Bold indicates statistical significance. SS = sums of squares.

	Main Effects	SS	df Num	df Den	F	Full p	27C & 30C p	D3 & D7 p
C1	**Temperature**	**10.32**	**2**	**12.006**	**20.91**	**<0.01**	**<0.01**	**<0.01**
	**Day**	**34.86**	**2**	**206.016**	**70.59**	**<0.01**	**<0.01**	**<0.01**
	**Infection Type**	**1.57**	**1**	**205.2**	**6.37**	**0.01**	**<0.01**	0.25
	Temperature:Day	0.56	2	206.016	1.14	0.32	0.34	0.20
	Temperature:Infection Type	0.49	2	205.797	1.00	0.37	0.18	0.32
	**Day:Infection Type**	**6.74**	**2**	**205.146**	**13.65**	**<0.01**	**<0.01**	**<0.01**
	Temperature:Day:Infection Type	0.17	2	205.146	0.34	0.72	0.73	0.29
D1	**Temperature**	**4.91**	**2**	**8.43**	**9.79**	**0.01**	0.22	**0.01**
	**Day**	**12.92**	**2**	**277.09**	**25.77**	**<0.01**	**<0.01**	**<0.01**
	Infection Type	0.93	1	278.98	3.71	0.06	**<0.01**	0.08
	**Temperature:Day**	**2.36**	**3**	**277.00**	**3.14**	**0.03**	**0.01**	**0.01**
	Temperature:Infection Type	0.42	2	278.69	0.84	0.43	0.35	0.55
	Day:Infection Type	0.92	2	278.66	1.84	0.16	0.07	0.07
	Temperature:Day:Infection Type	1.63	3	278.30	2.17	0.09	0.05	0.05
F1	Temperature	0.00	2	9.05	0.00	1.00	0.97	0.70
	**Day**	**8.06**	**2**	**219.03**	**13.24**	**<0.01**	**<0.01**	**<0.01**
	Infection Type	0.17	1	219.72	0.56	0.45	0.61	0.60
	Temperature:Day	1.77	3	218.33	1.94	0.12	0.07	0.28
	Temperature:Infection Type	0.03	2	219.27	0.04	0.96	0.85	0.48
	**Day:Infection Type**	**3.43**	**2**	**217.41**	**5.63**	**<0.01**	**0.02**	**<0.01**
	Temperature:Day:Infection Type	0.88	3	217.65	0.96	0.41	0.24	0.98
G3	Temperature	1.31	2	7.83	1.41	0.30	0.30	0.12
	**Day**	**24.95**	**2**	**327.13**	**26.77**	**<0.01**	**<0.01**	**<0.01**
	Infection Type	0.11	1	329.22	0.24	0.63	0.94	**0.01**
	Temperature:Day	3.77	3	327.16	2.70	0.05	0.06	0.22
	Temperature:Infection Type	0.51	2	328.87	0.55	0.58	0.30	0.79
	**Day:Infection Type**	**12.51**	**2**	**330.27**	**13.42**	**<0.01**	**<0.01**	**<0.01**
	Temperature:Day:Infection Type	1.85	3	330.61	1.32	0.27	0.61	0.15

Table S8. Three-way ANOVA linear mixed effects model summary table of the effect of day, temperature number of species infected per larva, and their interactions on the number of cells per larva. SS = sums of squares.

	SS	Num df	Den df	F	P
**Number of Species**	**44.049**	**4**	**181**	**44.3961**	**<0.01**
Day	0.815	2	181	1.6424	0.20
Temperature	1.335	2	181	2.6903	0.07
Number of Species:Day	0.909	7	181	0.5233	0.82
Number of Species:Temperature	1.535	6	181	1.0311	0.41
**Day:Temperature**	**2.167**	**3**	**181**	**2.9127**	**0.04**
Number of Species:Day:Temperature	1.651	7	181	0.9506	0.47

Table S9. Number of larvae in the 4-way choice treatment by temperature treatment and time point analyzed in the mixed assemblage dynamics models (only larvae that were infected are included). This data is visualized in Figure 4.

	Day 3	Day 7	Day 14
27ºC	41	23	22
30ºC	38	27	14
31ºC	36	7	NA

Table S10. Permutation test for homogeneity of multivariate dispersions for the 4-way choice treatment.

		df	SS	F	N.Perm	P
Day 3	Groups	2	0.02	0.43	999	0.64
	Residuals	112	2.67			
Day 7	Groups	2	0.03	1.16	999	0.33
	Residuals	54	0.79			
Day 14	Groups	1	0.02	0.95	999	0.35
	Residuals	34	0.79			

Table S11. (Table 4 with subset models). ANOVA table of mixed effect model analysis for the 4-way choice larvae of the effect of Symbiodiniaceae genus (*Cladocopium*, *Durusdinium*, *Fugacium* or *Gerakladium*), time (days), temperature (27, 30, and 31ºC), and their interactions on the relative abundances of symbiont cells larva^{-1}. Bold indicates statistical significance. SS = sums of squares.

					Full	27 & 30C	Days 3 & 7
Main Effects	SS	df Num	df Den	F	P	P	P
**Symbiont species**	**26.73**	**3**	**800**	**106.65**	**<0.01**	**<0.01**	**<0.01**
Day	0	2	800	0.02	0.98	0.94	0.84
Temperature	0.16	2	800	0.93	0.4	0.25	0.31
**Symbiont species:Day**	**2.49**	**6**	**800**	**4.96**	**<0.01**	**<0.01**	0.56
**Symbiont species:Temperature**	**6.06**	**6**	**800**	**12.08**	**<0.01**	**<0.01**	**<0.01**
Day:Temperature	0.02	3	800	0.06	0.98	0.93	0.96
Symbiont species:Day:Temperature	0.88	9	800	1.16	0.31	0.23	0.5

Table S12. Generalized mixed effects model (binomial) of survivorship of larvae by symbiont infection treatment group (no-choice *Cladocopium*, *Durusdinium*, *Fugacium*, and *Gerakladium,* and the 4-way choice), temperature treatment (27, 30 and 31ºC), day, and their interaction, with jar as a random effect.

Main Effects	Chisq	Df	Pr(>Chisq)
**Temperature**	**666.75**	**18**	**<0.01**
**Infection Treatment**	**134.58**	**22**	**<0.01**
**Day**	**909.4**	**14**	**<0.01**
**Infection Treatment**	**41.12**	**14**	**<0.01**
**Temp:Day**	**25.27**	**10**	**<0.01**
Infection Treatment:Day	12.53	14	0.56
Temperature:Infection Treatment:Day	3.29	16	1

Table S13. Mean larval survivorship (± standard error) for the five symbiont infection treatments (no-choice *Cladocopium*, *Durusdinium*, *Fugacium* and *Gerakladium*) and the 4-way choice treatment. Also included is the total survivorship for the aposymbiotic controls.

Temperature	Symbiont	Day	Mean (%)	se
27ºC	4-way choice	7	29.20	2.20
27ºC	4-way choice	14	9.07	1.57
27ºC	No-choice *Cladocopium*	7	26.80	2.08
27ºC	No-choice *Cladocopium*	14	9.73	0.81

27ºC	Aposymbiotic Control	7	20.80	NA
27ºC	Aposymbiotic Control	14	5.60	NA
27ºC	No-choice *Durusdinium*	7	26.80	3.45
27ºC	No-choice *Durusdinium*	14	6.13	0.87
27ºC	No-choice *Fugacium*	7	35.47	6.30
27ºC	No-choice *Fugacium*	14	12.40	3.49
27ºC	No-choice *Gerakladium*	7	30.80	7.11
27ºC	No-choice *Gerakladium*	14	10.67	2.54
30ºC	4-way choice	7	23.33	3.15
30ºC	4-way choice	14	4.24	0.40
30ºC	No-choice *Cladocopium*	7	18.18	1.60
30ºC	No-choice *Cladocopium*	14	3.33	1.09
30ºC	Aposymbiotic Control	7	5.00	NA
30ºC	Aposymbiotic Control	14	2.27	NA
30ºC	No-choice *Durusdinium*	7	15.00	1.36
30ºC	No-choice *Durusdinium*	14	1.67	0.15
30ºC	No-choice *Fugacium*	7	31.21	6.90
30ºC	No-choice *Fugacium*	14	5.76	1.49
30ºC	No-choice *Gerakladium*	7	24.39	4.02
30ºC	No-choice *Gerakladium*	14	5.91	1.31
31ºC	4-way choice	7	7.11	4.89
31ºC	4-way choice	14	0.00	0.00
31ºC	No-choice *Cladocopium*	7	9.19	1.57
31ºC	No-choice *Cladocopium*	14	2.37	0.15
31ºC	Aposymbiotic Control	7	7.11	NA
31ºC	Aposymbiotic Control	14	2.22	NA
31ºC	No-choice *Durusdinium*	7	4.30	0.74

31ºC	No-choice *Durusdinium*	14	0.00	0.00
31ºC	No-choice *Fugacium*	7	7.41	2.52
31ºC	No-choice *Fugacium*	14	0.74	0.30
31ºC	No-choice *Gerakladium*	7	9.63	3.86
31ºC	No-choice *Gerakladium*	14	1.78	0.44
